# Flash Communication: Siloxides Promote the Iron-Catalyzed Borylation of Aryl Chlorides

**DOI:** 10.1021/acs.organomet.6c00235

**Published:** 2026-08-14

**Authors:** Patrick Daly-Dee, Luke Davies, Graham J. Tizzard, Simon J. Coles, Sebastien Monfette, Robin B. Bedford

**Affiliations:** † School of Chemistry, 1980 University of Bristol, Cantock’s Close, Bristol BS8 1TS, U.K.; ‡ EPSRC National Crystallography Service, School of Chemistry and Chemical Engineering, 7423 University of Southampton, Southampton SO17 1BJ, U.K.; § Pfizer Chemical Research and Development, Pfizer Inc., Groton, Connecticut 06340, United States

## Abstract

In contrast to alkoxide salts, siloxide salts MOSiR_3_ (M = Na, Li) can be used to good effect as activators for B_2_pin_2_ in the iron-catalyzed borylation of aryl chloride substrates, in many cases giving results comparable to or better than those obtained previously with highly pyrophoric *^t^* BuLi acting as an activator. Preliminary mechanistic investigations reveal a protracted induction period, most likely associated with precatalyst reduction, and these also suggest that the NaOSi*^t^* BuMe-activated B_2_pin_2_ is not as reducing as the equivalent *^t^* BuLi-activated species.

## Graphical abstract

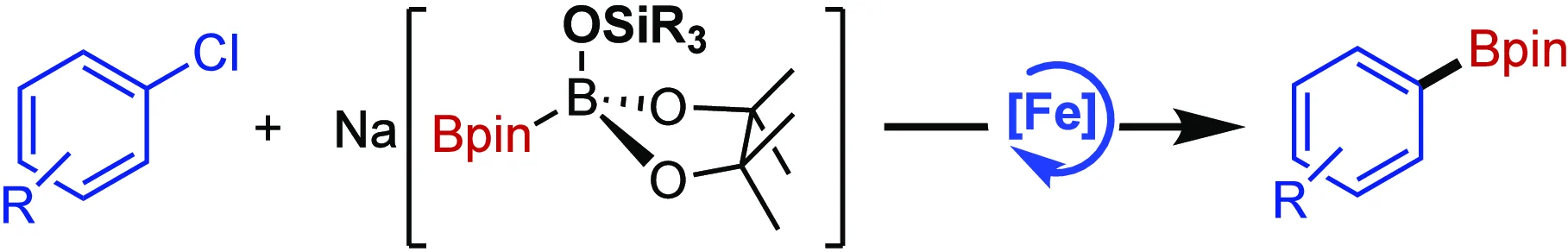

The transition metal catalyzed borylation of aryl halides, first described by Miyaura,^1^ is a powerful method for the formation of aryl boronic esters. While palladium catalysis dominates Miyaura borylation of aryl electrophiles,^2–5^ catalysis based on more Earth-abundant elements such as copper,^6–8^ nickel,^9–14^ cobalt^15,16^ and zinc^17,18^ have also been shown to be effective. In this regard, iron represents an ideal replacement for palladium, and iron-catalyzed borylation of aryl electrophiles has been demonstrated with aryl diazonium salts,^19^ pyridone ethers,^20^ carboxylic acids^21^ and aryl fluorides.^22,23^ We recently showed that aryl chlorides and triflates could be readily exploited as substrates when Li­[B_2_pin_2_-^t^Bu], **1a**, was used as the borylating reagent.^24,25^ Unfortunately, the requirement of highly pyrophoric *^t^* BuLi in the preparation of **1a** limits the synthetic appeal of this transformation and prompted an investigation of alternative reagents for activating B_2_pin_2_. Nakamura has shown that KO*^t^* Bu can be exploited with certain aryl halide substrates,^26^ but good activity is largely limited to fused-ring and 4-aryl-substituted aryl substrates.^27^ Here we report the somewhat surprising finding that, despite their reduced basicity compared with alkoxides,^28^ the simple siloxide salts NaOSiR_3_ effectively activate B_2_pin_2_ for iron-catalyzed borylation of aryl chlorides.

A survey of diboron esters (B_2_pin_2_, B_2_neop_2_, and B_2_eg_2_) with various activating agents (organolithiums, Grignard reagents, alkoxide salts, and LiHMDS) in the borylation of chlorobenzene (**2a)** catalyzed by a mixture of Fe­(OTf)_2_/IMes under our previously optimized conditions^25^ (Supporting Information Figure S2) revealed that, aside from the *^t^* BuLi and *^s^* BuLi adducts of B_2_pin_2_, most of the activated boronates did not lead to productive catalysis; however, the NaO*^t^* Bu adduct of B_2_pin_2_ (**1b)** offered a promising 24% yield. Replacing NaO*^t^* Bu with NaOSiMe_3_ gave the analogous salt **1c** *in situ*, which in turn led to a noticeable improvement in the borylation of **2a** giving the desired arylboron ester **3a** in 51% yield, suggesting that p*K*_a_ alone is not a useful metric in the selection of activating reagents. Subsequent screening of siloxide salts prepared in situ, without isolation or further purification, from the parent silanol and Li, Na, or KHMDS in the borylation of **2a** revealed a potential trend of increased donor ability of the substituents giving higher yields with the lithium and sodium salts investigated (Supporting Information Table S6). In contrast, the potassium siloxides offered poor performance. Following further optimization, targeted primarily at minimizing side reactions (see the Supporting Information for details), NaOSi^t^BuMe_2_ was identified as the optimal activator, and the resultant activated boron reagent, **1d** (formed in situ) was carried forward in a brief investigation of the effect of precatalyst structure on activity (Figure 1). Early on in these studies, we found that, in contrast to the previously reported conditions with ^t^BuLi acting as the activator,^25^ the use of MgBr_2_ as an additive was detrimental to performance (Supporting Information Table S8), and this salt was therefore omitted from subsequent reactions.

**Figure 1:**
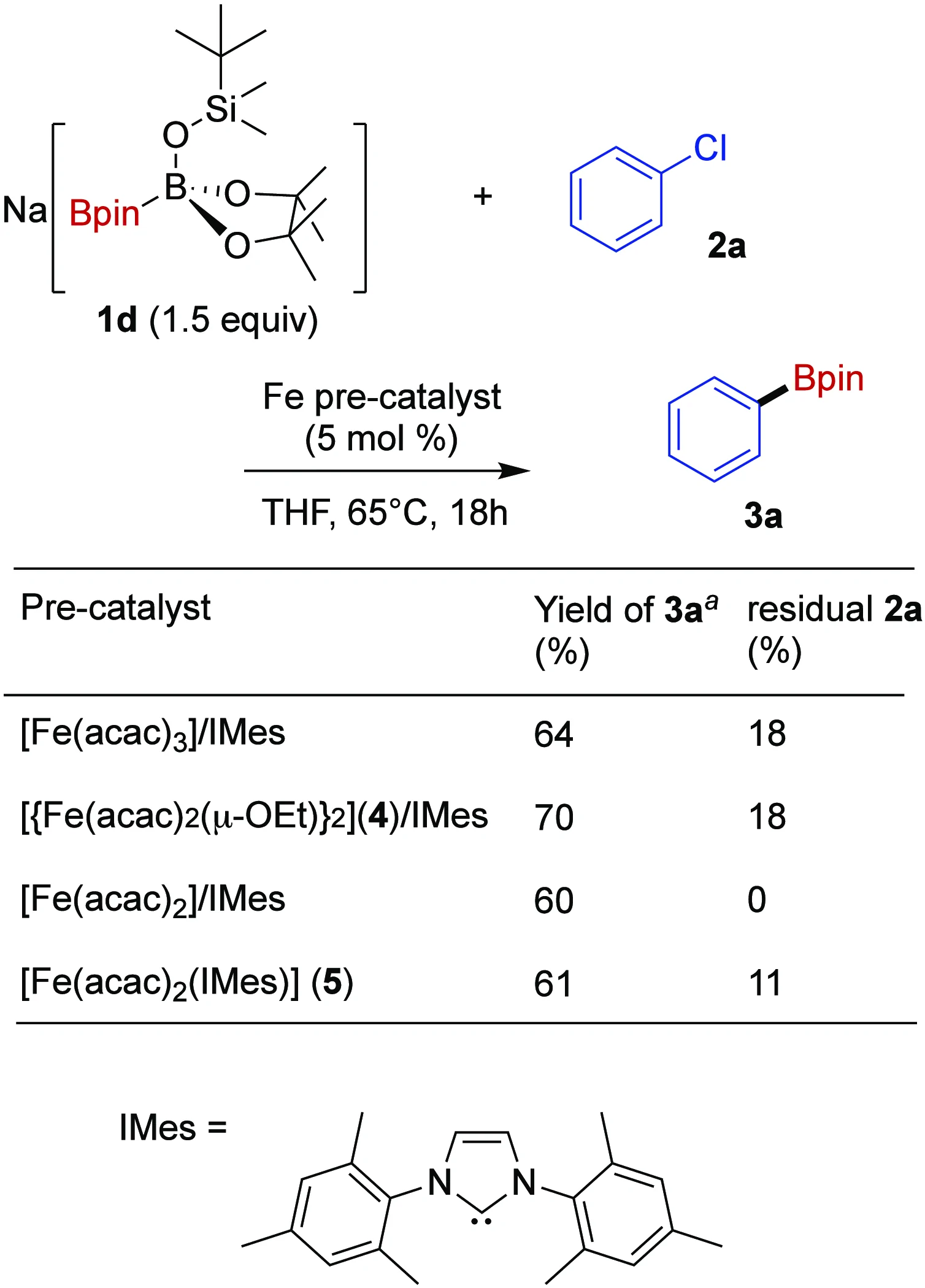
Borylation of chlorobenzene (**2a**) with **1d** (formed in situ) catalyzed by iron-acac/IMes precatalysts. Conditions: Fe source (0.0125 mmol of Fe) and IMes (0 or 0.0125 mmol); THF (1 mL); 15 min at room temperature; **2a** (0.25 mmol); B_2_pin_2_ (0.375 mmol 0.75 M THF solution); NaOTBDMS (0.375 mmol, from a preformed stock solution of NaHMDS and ^t^BuMe_2_SiOH, THF, 1 mL); 65 °C for 18 h. *^a^* Yield determined by GC (dodecane internal standard).

From the data in Figure 1 it can be seen that Fe­(III) precursors perform marginally better than Fe­(II) analogues. During these investigations we found somewhat frustratingly that no commercial sources of [Fe­(acac)_2_] that we examined actually proved to be the claimed material; instead, they all consisted of [Fe­(acac)_3_] as determined by ^1^H NMR spectroscopic investigation. The very facile conversion of the Fe­(II) to the Fe­(III) complex has been noted previously.^29^ Instead, we prepared genuine samples of [Fe­(acac)_2_] by a literature method,^30^ and advise similar caution to users of commercially sourced material. While the preformed Fe­(II) complex [Fe­(acac)_2_(IMes)], **5**, showed marginally poorer activity than the Fe­(III)/IMes mixtures, it proved to be synthetically far more tractable as a single component precatalyst: attempts to isolate Fe­(III) analogues have so far been unsuccessful. Thus, reaction of [Fe­(acac)_2_] with either free IMes or IMesH.Cl and NaHMDS in THF provided **5** in good yield as a yellow-brown paramagnetic solid with a magnetic moment of 4.6 μ_B_ recorded in solution at room temperature (Evans’ method),^31,32^ consistent with a high-spin (*S* = 2) ground state. Figure 2(a) shows the single crystal molecular structure of **5** while Figure 2(b) shows the DFT-calculated^33^ high-spin (*S* = 2) structure (PBE0-d3bj/def2-SVP­(def2-TZVP on Fe)), with both displaying a similar geometry at iron, intermediate between square-based pyramidal and trigonal bipyramidal (τ_5_ parameter^34^ determined from the crystal structure = 0.44; determined from the HS DFT structure = 0.61). The bond metrics associated with the donor atoms and the Fe center determined in the crystal structure are well reproduced in the DFT model with mean absolute errors of 0.0033 Å and 0.25° for the calculated bond lengths and angles, respectively. Calculation of free energy for the optimized structures (PBE0-d3bj/def2-TZVP//PBE0-d3bj/def2-SVP­(def2-TZVP on Fe)) with low (*S* = 0) and intermediate (*S* = 1) spin-states indicate they are disfavored by 32.8 and 21.8 kcal/mol respectively, compared with the high-spin state, consistent with the magnetic data obtained above.

**Figure 2:**
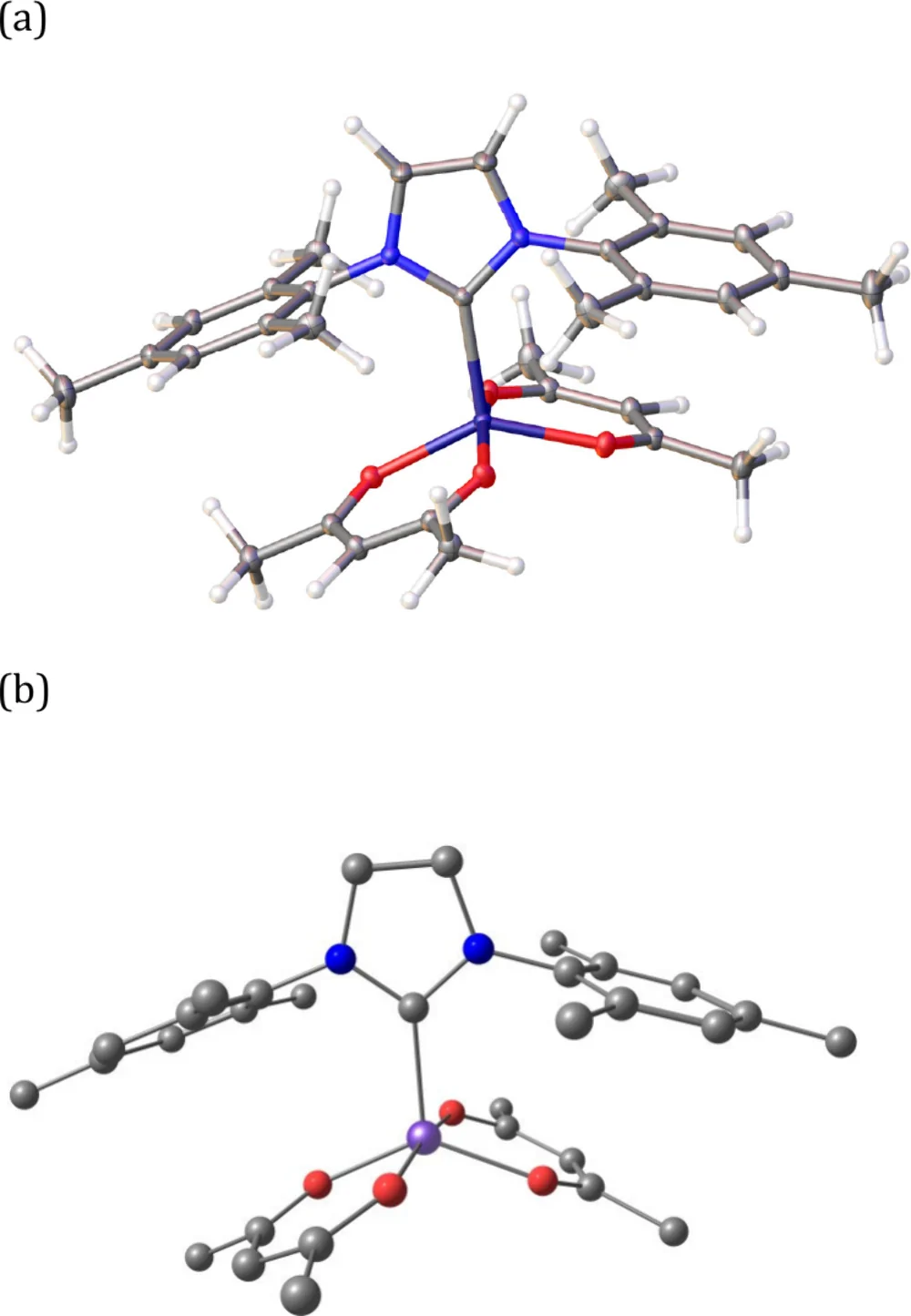
(a) Single crystal X-ray structure of complex **5** and (b) DFT-calculated high-spin structure of **5** (PBE0-d3bj/def2-SVP­(def2-TZVP on Fe), hydrogen atoms removed for clarity.

Final catalytic optimization focused on changing solvent and reaction time (Supplementary Tables S11 and S12), with the former showing all changes away from THF to be deleterious and the latter indicating a reaction time of 40 h gave improved yield of the desired product, suggesting slow catalysis but with reasonably stable active species. With the optimized conditions in hand, we undertook a brief substrate scope investigation with the **4**/IMes mixture employed as precatalyst and the results of this study are summarized in Figure 3 and Supplementary Figure S5 (unsuccessful reactions). The desired products **3a**–**e** were formed in better spectroscopic yield (>10% increase in relative yield) than previously obtained using ^t^BuLi as the activating agent for B_2_pin_2_,^25^ with a particularly notable improvement for the electron-deficient products **3c** and **d**. By contrast, products **3f**–**h** were obtained in very similar yield regardless of the activator employed, while products **3i**–**m** were furnished in significantly poorer yield (>10% decrease in relative yield) when the siloxide activator was employed. In contrast to the reaction with B_2_pin_2_ activated by ^t^BuLi, the ethylcarboxylate function was not tolerated (Supplementary Figure S6); however, the bulkier *tert*-butylcarboxylate was, furnishing **3o** in modest yield. Acetal protection of a ketone and *N*-alkylation of an indole allowed the products **3p** and **3q** to be obtained, whereas the unprotected functions were not tolerated (Supplementary Figure S5). In contrast to the result obtained in the formation of **3m**, most heterocyclic substrates were not tolerated (Supplementary Figure S5), nor were alkyl halide substrates, in sharp contrast to reactions employing ^t^BuLi as an activator for the B_2_pin_2_.^24^

**Figure 3:**
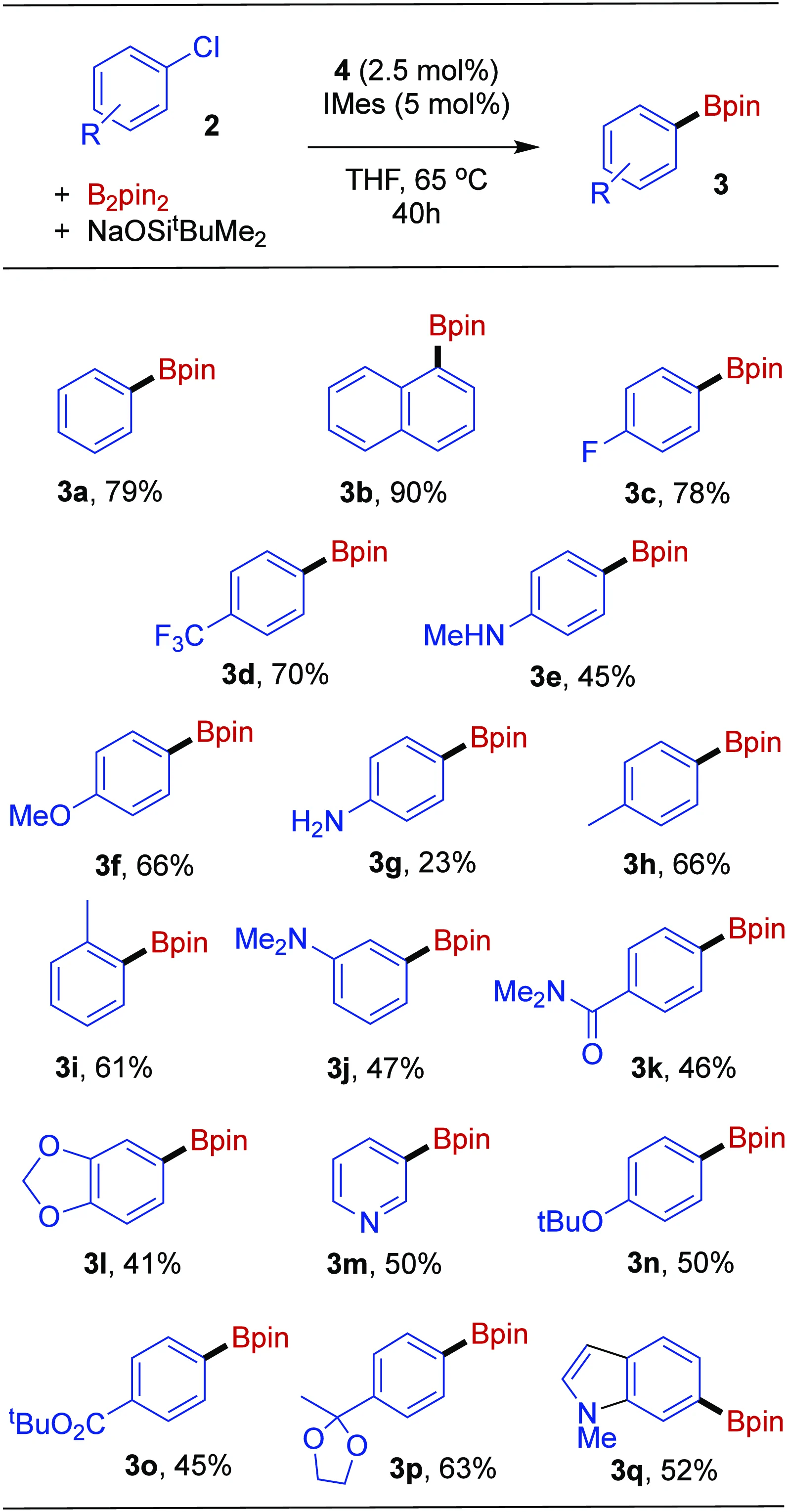
Borylation of aryl chloride substrates. Conditions: **4** (0.0063 mmol); IMes (0.0125 mmol); THF (1 mL); 15 min room temperature; **2** (0.25 mmol); B_2_pin_2_ (0.375 mmol, 0.75 M in THF); NaOTBDMS (0.375 mmol) from preformed stock solution of NaHMDS and tBuMe2SiOH in THF (1 mL); 65 °C for 40 h. Yield of **3a** determined by GC (dodecane internal standard). Spectroscopic yields of **3b**–**3q** determined by ^1^H NMR spectroscopy (1,3,5-trimethoxybenzene internal standard).

Summarizing the catalytic data, there are considerable variations in performance from our previously reported system using ^t^BuLi as the activator for B_2_pin_2_, which may indicate distinct catalytic manifolds in operation. Full mechanistic investigations are beyond the scope of this preliminary study, but we note that there is a considerable induction period in the catalytic reaction of about 3 h (Supplementary Figure S7). We suspect that this period is associated with precatalyst reduction, indeed, a reaction mixture in which the **3a** is left out and then added after 3 h shows rapid onset of turnover (Supplementary Figure S6). In similar iron-catalyzed transformations, with a reductive induction period, we have used the formation of byproducts produced by the homocoupling of the nucleophile as a proxy for the bulk oxidation state of the iron formed under kinetically relevant conditions.^35–37^ Here this is more challenging as the byproduct would be B_2_pin_2_, a substrate for the reaction. To circumvent this issue we attempted crossover experiments with B_2_pin_2_-*d12* and B_2_pin_2_. Unfortunately, while crossover was observed (see Supporting Information for details), this was also the case in the absence of the iron precatalyst, negating the utility of this approach.

Turning to the reducing abilities of the boronates **1b** and **1d**, we find that in the former case, where the B_2_pin_2_ is activated by *^t^* BuLi, the Fe­(II)/IMes precatalyst explored previously^25^ can be reduced to an Fe(0) species which could be ‘trapped’ in the presence of the diene 1,3-divinyltetramethyldisiloxane (dvtms), giving the previously reported complex [Fe­(IMes)­(dvtms)], **6** ^37,38^ as observed by ^1^H NMR spectroscopy (Supplementary Figure S10). By contrast, **6** was not observed when the B_2_pin_2_ was activated with NaOSi^t^BuMe_2_. Instead, a new species, **7**, was observed in the ^1^H NMR spectrum (Supplementary Figure S11) that was also observed in the absence of the dvtms (Figure S12). Attempts to isolate and characterize **7** are ongoing.

Finally, radical trap experiments did not indicate the intermediacy of radicals during turnover (see Supporting Information for full details). For instance, while TEMPO switched off the catalytic reaction, no adducts with radicals derived from either substrate were observed in the resultant reaction mixture. Meanwhile, the addition of 1,1-diphenylethene did not impact activity, and no radical-derived adducts were observed.

In summary, we have shown that the far more readily handled alkylsiloxide salts such as NaOSi^t^BuMe_2_ can be used in place of ^t^BuLi as an activating agent for B_2_pin_2_ in the iron-catalyzed borylation of aryl chlorides. While there are many similarities in both the performance and preliminary mechanistic observations between the two systems, there are also distinct and intriguing differences that prompt further, ongoing investigations.

## Supplementary Material





## Data Availability

^1^H NMR data used to determine the yields in Figure 3 and Supplementary Figure S6 are available at the University of Bristol data repository, data.bris, at 10.5523/bris.170r4mfzxrnut29c0xoqgfr08h. All other data underlying this study are available in the published article and its Supporting Information.

## Notes

Cartesian coordinates for **5** calculated in high-, intermediate- and low-spin states (XYZ)
Experimental details and spectroscopic data (PDF)
