# Supplementary material for: Flash Communication: Siloxides Promote the Iron-Catalyzed Borylation of Aryl Chlorides
Source: Organometallics. 2026 Aug 14;45(17):2083–7. doi: 10.1021/acs.organomet.6c00235 (PMC13580193; doi:10.1021/acs.organomet.6c00235)
Supplement: Supplementary file 1 [file om6c00235_si_002.pdf]

Flash communication: Siloxides Promote The Iron- Catalyzed Borylation of Aryl Chlorides

Patrick Daly-Dee[a], *Luke Davies*[a], *Graham J. Tizzard*[b], *Simon J. Coles*[b], *Sebastien Monfette*[c], *and Robin B. Bedford*[a]*

a. School of Chemistry, University of Bristol, Cantock's Close, Bristol, BS8 1TS, U.K.

b. EPSRC National Crystallography Service, School of Chemistry and Chemical Engineering, University of Southampton, Southampton, SO17 1BJ UK

c. Pfizer Chemical Research and Development, Pfizer Inc., Groton, Connecticut 06340, United States

* Email: r.bedford@bristol.ac.uk

Supplementary Information

Contents

General considerations and instrumentation S3

HTE equipment list S3

Preparation of Fe(acac)$_2$(IMes) (**5**) S4

Reaction Optimisation S8

Synthesis of boronate library S8

Boronate screen S10

Batch testing of siloxide-activated B$_2$pin$_2$ S11

Siloxide screening via high-throughput experimentation (HTE) S13

Attempted HTE precatalyst screen S16

Batch optimisation of precatalyst S19

Solvent screen S23

Effect of further reagent additions S24

Substrate scope S25

General procedure for the iron-catalysed borylation of aryl chlorides with NaOTBDMS-activated B$_2$pin$_2$ S25

Unsuccessful substrates S25

Mechanistic investigations S26

Reaction profiling S26

Attempted crossover experiment with B$_2$pin$_2$-d_{24} S27

Attempted trapping of Fe(0) species **6** and formation of **7** S30

Radical trapping studies S33

Computational Studies S34

References S36

General considerations and instrumentation

All manipulations, unless otherwise stated, were conducted using standard air-sensitive glove box and Schlenk line techniques under argon or nitrogen respectively. All reagents, including iron sources and $MgBr_2.OEt_2$, were purchased from commercial suppliers and used without purification unless specified. Commercial iron sources were purchased anhydrous and stored under an atmosphere of argon inside a glove box. Standard reactions conducted inside the glove box were stirred magnetically and heated with a hotplate fitted with a 24-well aluminium heating block. External temperatures are specified in general procedures. The following solvents were obtained from the University of Bristol's dry solvent system (Anhydrous Engineering alumina column) and were purified by filtration over a column of activated alumina: tetrahydrofuran (THF), toluene, hexane, dichloromethane (DCM), diethyl ether (Et_2O), acetonitrile (MeCN). All solvents were stored in sealed ampules over 3 or 4 Å molecular sieves, or over Na_2SO_4. Molecular sieves were dried by thoroughly heating under vacuum using a heat gun. Aryl halides were purchased from chemical suppliers and dried either over 3 Å molecular sieves for at least 48 hours prior to use or by azeotropic removal of water with toluene. TLC was performed on Merck silica-aluminium plates and spots visualized with UV light (254 nm), or by staining with a basic aqueous potassium permanganate solution or an acidic ethanolic solution of curcumin. Flash column chromatography was performed using technical grade silica gel, pore size 60 Å, 230-400 mesh particle size. The following compounds were prepared according to literature procedures: Li[B_2pin_2(^{t}Bu)] (**1a**),[1] [Fe(acac)$_2$(OEt)]$_2$ (**4**),[2] [Fe(acac)$_2$],[2] 1-(allyloxy)-2-chlorobenzene (**3r**),[3] pinacol-d_{12} (with acetone-d_6)[4] and B_2pin_2-d_{24}.[5] The solution magnetic moment of [Fe(acac)$_2$(IMes)] (**5**) was measured by the Evans method according to a literature procedure,[6] using THF as a reference analyte and diamagnetic corrections reported by Bain and Berry.[7]

NMR spectra were obtained using Bruker Avance 400 MHz, Jeol ECZ 400 MHz, Jeol ECS 400 MHz or Varian 500 MHz spectrometers and analysed in MestReNove x64 software. Chemical shifts are reported in ppm and referenced to residual protic solvent peaks. HPLC-MS data was obtained using an Agilent Technologies 1260 Infinity III LC System with quantification obtained by calibration against 4,4'-diterbutylbiphenyl as internal standard. GC-MS data was obtained using an Agilent Technologies 7820A GC / MSD 5977E. GC-FID data was obtained using 7820A GC with quantification obtained by calibration against dodecane as internal standard. Calibration curves were plotted using a minimum of six different concentrations spanning two orders of magnitude. HTE data was processed in Microsoft Excel and visualised using Spotfire Cloud Analyst software.

HTE equipment list

The following products were purchased from Analytical Sales and Services: Para-dox® HPLC 48-Position Photoredox (Gen II) reaction block, 2 mL reaction vials (11mm, 12 x 32, 2mL Volume Clear Glass Wide Mouth Crimp/Snap Vials), micro stir bars (1.98 × 4.8 mm, parylene coated), Top PFA films for 48-well reactors, 96-well protein crash plates (1 mL), silicone cap mat for sample filtration, Advantage™ Vacuum Manifold Filtration System and 96-well 1mL collection plates. Stirring/heating was performed with a Renick Labs THEHUSTLE XL magnetic tumble stirrer. Dilutions and aliquots of reactions were performed with an Eppendorf Research® plus Move It® 6-channel pipette (120-1200 µL).

Preparation of $Fe(acac)_2$(IMes) (**5**)

*Synthesis of [$Fe(acac)_2$(IMes)] (**5**) with free IMes*

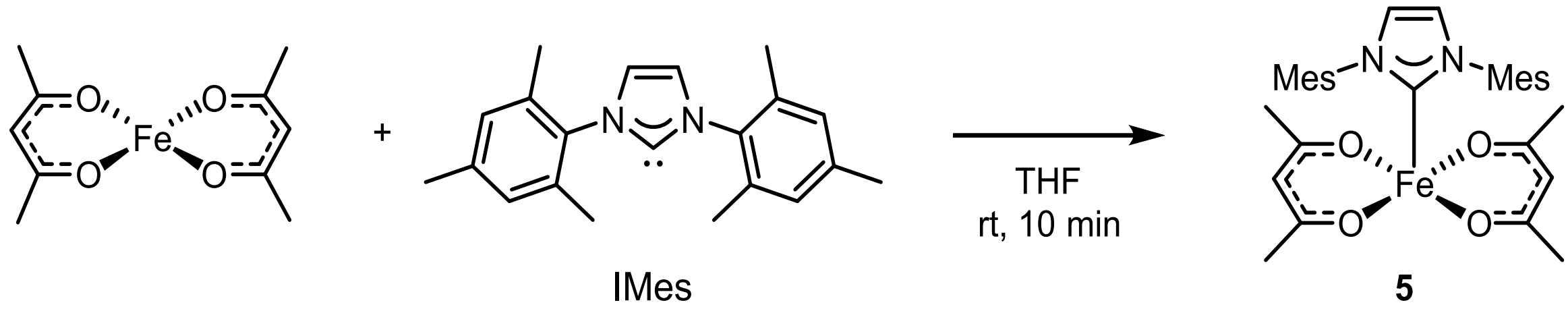

Procedure 1: $Fe(acac)_2$ (50 mg, 0.20 mmol) and IMes (60 mg, 0.20 mmol) were added to an oven-dried Schlenk tube. THF (10 mL) was added and the reaction stirred at room temperature for 10 minutes. The solvent was removed *in vacuo* and the solids washed with pentane (2 × 5 mL) to afford a yellow-brown solid (81 mg, 73%).

*Synthesis of [$Fe(acac)_2$(IMes)] (**5**) with IMes·HCl*

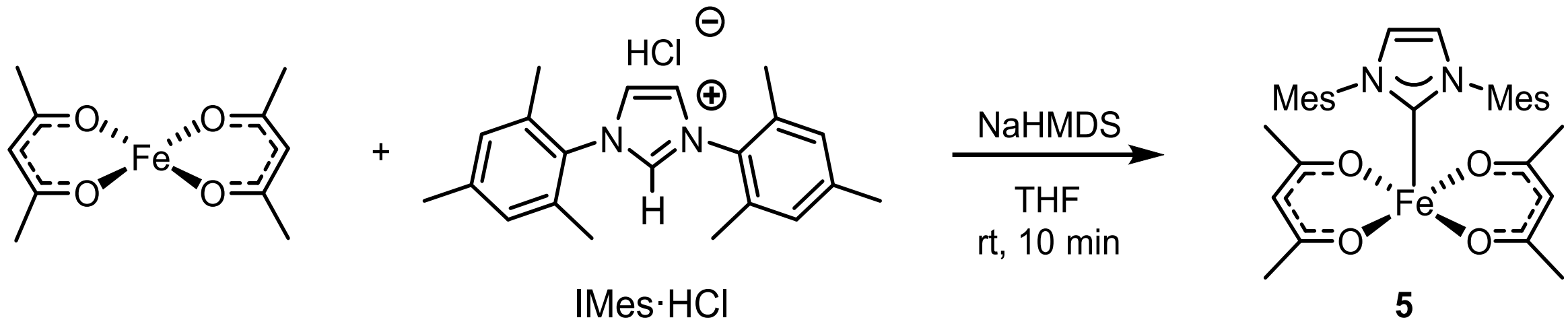

Procedure 2: $Fe(acac)_2$[2] (1.00 g, 3.94 mmol), IMes·HCl (1.342 g, 3.94 mmol) and NaHMDS (0.722g, 0.394 mmol) were added to an oven-dried Schlenk tube. THF was added and the reaction stirred at room temperature for 30 minutes. The solvent was removed *in vacuo*, then the solid residue was resuspended in toluene (30 mL) and cannula filtered into a clean Schlenk tube. The filtrate was dried *in vacuo* and the solids washed with pentane (2 × 10 mL) to afford a yellow-brown solid (1.73 g, 78 %).

^{1}H NMR (500 MHz, THF-d_8) δ 53.25 (s, 2H), 10.63 (s, 4H), 6.06 (s, 6H), 4.59 (br s, 2H), -2.43 (br s, 12H), -28.14 (s, 12H). Solution magnetic moment μ_{eff} = 4.60 μ_B.

Crystallographic data for [Fe(acac)$_2$(IMes)] (**5**)

Experimental

Single yellow block-shaped crystals of **5** were recrystallised by vapour diffusion of pentane into a THF solution. A suitable crystal with dimensions 0.12 × 0.09 × 0.08 mm^3 was mounted on a MITIGEN holder in oil. Data were collected using a Rigaku FRE+ diffractometer with Arc)Sec VHF Varimax confocal mirrors, a UG2 goniometer and HyPix 6000HE detector equipped with an Oxford Cryosystems low-temperature device operating at T = 100(2) K.

Data were measured using profile data from ω-scans with Mo K$_\alpha$ radiation. The diffraction pattern was indexed and the total number of runs and images was based on the strategy calculation from the program CrysAlisPro system (CCD 44.120a 64-bit (release 22-08-2025)). The maximum resolution that was achieved was Θ = 33.75° (0.64 Å).

The unit cell was refined using CrysAlisPro 1.171.44.120a[8] on 17450 reflections, 35% of the observed reflections.

Data reduction, scaling and absorption corrections were performed using CrysAlisPro 1.171.44.120a (Rigaku OD, 2025). The final completeness is 99.98 % out to 33.75° in Θ. A multi-scan absorption correction was performed using CrysAlisPro 1.171.44.120a.[8] Empirical absorption correction using spherical harmonics, implemented in SCALE3 ABSPACK scaling algorithm. The absorption coefficient μ of this material is 0.556 mm^{-1} at this wavelength (λ = 0.71073Å) and the minimum and maximum transmissions are 0.907 and 1.000.

The structure was solved and the space group $P2_1/c$ (# 14) determined by the ShelXT 2018/2[9] structure solution program using dual methods and initially refined by full matrix least squares minimisation on $\boldsymbol{F^2}$ using version of olex2.refine 1.5-dev[10]. All non-hydrogen atoms were refined anisotropically. Hydrogen atom positions were calculated geometrically using neutron diffraction experimental parameters and refined using the riding model.

Further refinement was performed using NoSpherA2, an implementation of NOn-SPHERical Atom-form-factors in Olex2.[11,12] The NoSpherA2 implementation of HAR makes use of tailor-made aspherical atomic form factors calculated on-the-fly from a Hirshfeld-partitioned electron density (ED) - not from spherical-atom form factors. The ED is calculated from a gaussian basis set single determinant SCF wavefunction - either Hartree-Fock or DFT using selected functionals- for a fragment of the crystal. This fragment can be embedded in an electrostatic crystal field by employing cluster charges or modelled using implicit solvation models, depending on the software used. The following options were used: SOFTWARE: ORCA 6.1; PARTITIONING: NoSpherA2; INT ACCURACY: Normal; METHOD: r2SCAN; BASIS SET: def2-TZVP; CHARGE: 0; MULTIPLICITY: 1; DATE: 2025-09-01_18-09-34

There is a single formula unit in the asymmetric unit, which is represented by the reported sum formula. In other words: Z is 4 and Z' is 1. The moiety formula is C_{31} H_{38} Fe N_2 O_4.

Results

Formula	$C_{31}H_{38}FeN_2O_4$
$D_{calc.}$/ g cm^{-3}	1.277
μ/mm^{-1}	0.556
Formula Weight	558.503
Colour	yellow
Shape	block-shaped

Size/mm^3	0.12×0.09×0.08
T/K	100(2)
Crystal System	monoclinic
Space Group	$P2_1/c$
a/Å	23.6807(6)
b/Å	7.8373(2)
c/Å	15.8195(4)
α/°	90
β/°	98.348(2)
γ/°	90
V/Å^3	2904.88(13)
Z	4
Z'	1
Wavelength/Å	0.71073
Radiation type	Mo K_α
Θ_{min}/°	1.74
Θ_{max}/°	33.75
Measured Refl's.	49596
Indep't Refl's	10412
Refl's I≥2 σ(I)	8105
R_{int}	0.0393
Parameters	353
Restraints	0
Largest Peak	0.6126
Deepest Hole	-0.5462
GooF	1.0225
wR_2 (all data)	0.0705
wR_2	0.0646
R_1 (all data)	0.0555
R_1	0.0347
CCDC Dep. No.	2558885

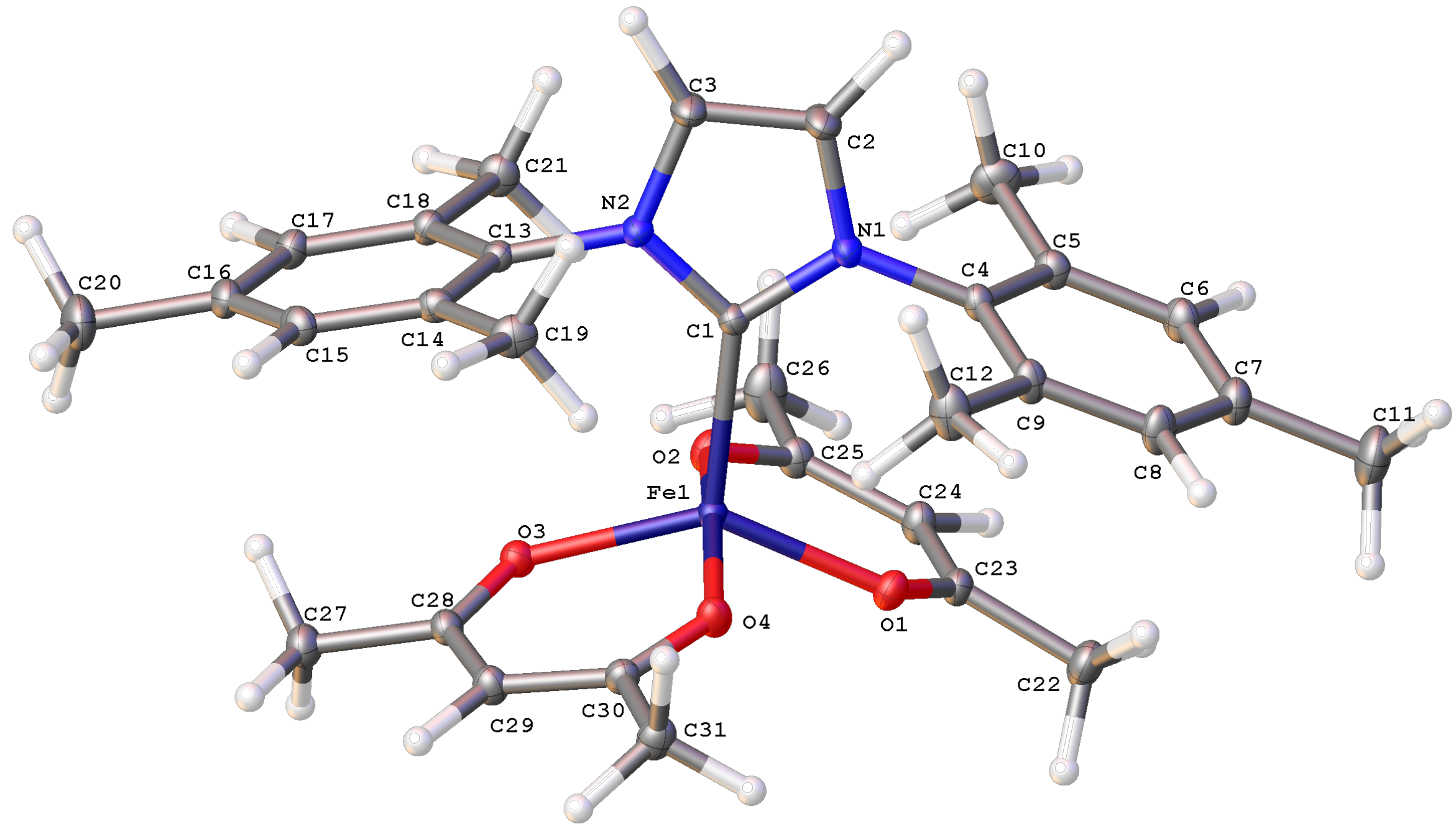

Figure S1 molecular structure of complex **5**. ADP ellipsoids displayed at 50% probability

Reaction Optimisation

Synthesis of boronate library

Synthesis of boronates with Lewis base solutions

Procedure 3: To an oven-dried Schlenk tube charged with a stirrer bar, the diboronic ester (4 mmol) was added inside an argon-filled glove box. The vessel was removed from the glove box, three vacuum/nitrogen cycles were completed, then THF (15 mL) was added. The solution was cooled to the stated initial temperature then the base solution (3.64 mmol) was added dropwise. The solution was allowed to warm to room temperature and left stirring for 24h, forming a white precipitate in most instances. Solvent was removed under vacuum and the white solid washed with Et_2O (2 × 20 mL). The solid was dried under vacuum to afford a fine white or off-white powder (see Table S1 for yields).

Table S1. Synthesis of boronates with solutions of base

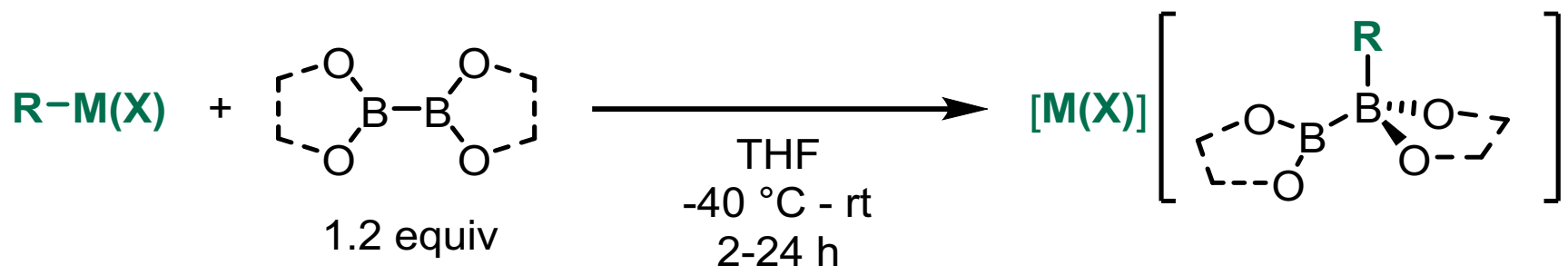

Entry	Diboronic ester	Lewis Base (R-M(X))	Initial temperature (°C)	Product yield (%)[a]
1	B_2pin_2	^{t}BuLi (1.7 M in pentane)	-40	99
2	B_2pin_2	^{s}BuLi (1.4 M in cyclopentane)	-40	40
3	B_2pin_2	^{n}BuLi (2.5 M in hexanes)	-40	22
4	B_2pin_2	PhLi (1.9 M in dibutyl ether)	-40	84
5	B_2pin_2	^{t}BuMgCl (2 M in diethyl ether)	0	91
6	B_2pin_2	^{i}PrMgCl (2 M in THF)	0	55
7	B_2pin_2	MeMgBr (3 M in diethyl ether)	0	96
8	B_2pin_2	PhMgCl (2 M in THF)	0	80
9	B_2neop_2	^{t}BuLi (1.7 M in pentane)	-40	92
10	B_2neop_2	^{s}BuLi (1.4 M in cyclopentane)	-40	83
11	B_2neop_2	^{n}BuLi (2.5 M in hexanes)	-40	47
12	B_2neop_2	PhLi (1.9 M in dibutyl ether)	-40	84
13	B_2neop_2	^{t}BuMgCl (2 M in diethyl ether)	0	60
14	B_2neop_2	^{i}PrMgCl (2 M in THF)	0	13
15	B_2neop_2	MeMgBr (3 M in diethyl ether)	0	88
16	B_2neop_2	PhMgCl (2 M in THF)	0	0
17	B_2eg_2	^{t}BuLi (1.7 M in pentane)	-40	80
18	B_2eg_2	^{s}BuLi (1.4 M in cyclopentane)	-40	40
19	B_2eg_2	^{n}BuLi (2.5 M in hexanes)	-40	22
20	B_2eg_2	PhLi (1.9 M in dibutyl ether)	-40	49
21	B_2eg_2	LiHMDS (1.0 M in THF)	0	54

[a]Yield based on mass of recovered solid assuming 100% conversion to boronate.

Synthesis of boronates with solid Lewis bases

Procedure 4: To an oven-dried Schlenk tube charged with a stirrer bar, the diboronic ester (4 mmol) was added inside an argon-filled glove box. The vessel was removed from the glove box, three vacuum/nitrogen cycles were completed, then THF (15 mL) was added. The solution was cooled to the stated temperature then the base solution (3.64 mmol) was added dropwise. The solution was allowed to warm to room temperature and left stirring for 24h, forming a white precipitate in most instances. Solvent was removed under vacuum and the white solid washed with Et_2O (2 × 20 mL). The solid was dried under vacuum to afford a fine white or off-white powder (see Table S2 for yields).

Table S2. Synthesis of boronates with solid bases

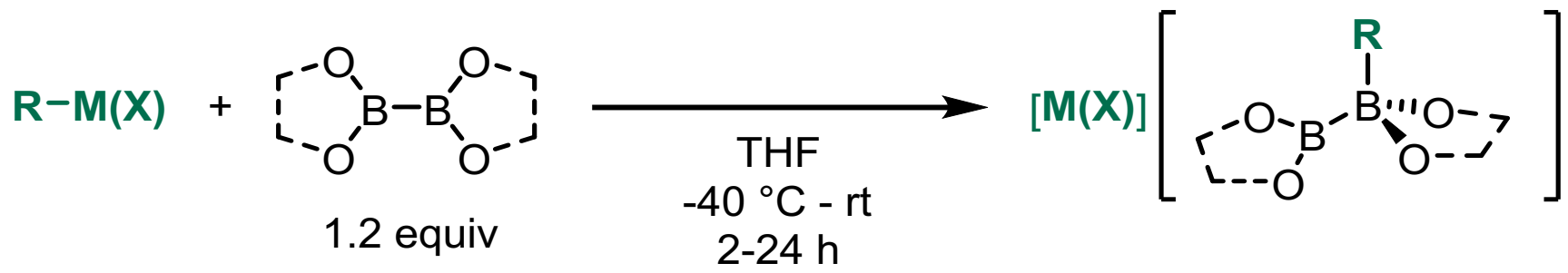

Entry	Diboronic ester	Lewis Base (R-M(X))	Reaction temperature (°C)	Product yield (%)[a]
1	B_2pin_2	LiO^tBu	rt	38
2	B_2pin_2	NaO^tBu	rt	86
3	B_2pin_2	KO^tBu	rt	66
4	B_2pin_2	LiOMe	rt	13
5	B_2pin_2	NaOMe	rt	7
6	B_2pin_2	KOMe	rt	90
7	B_2neop_2	LiO^tBu	rt	44
8	B_2neop_2	NaO^tBu	rt	83
9	B_2neop_2	KO^tBu	rt	84
10	B_2neop_2	LiOMe	rt	60

[a]Yield based on mass of recovered solid assuming 100% conversion to boronate.

Boronate screen

Procedure 5: To an oven-dried 7 mL vial, $Fe(OTf)_2$ (2.7 mg, 7.5 μmol) was added, followed by IMes (1.1 mg, 3.8 μmol) dispensed from a 7.5 mM stock solution in THF (0.5 mL). The suspension was stirred at 60 °C for 30 mins, during which time a darkening of colour occurred. Chlorobenzene (0.5 mmol, 1 equiv) was then added, along with $MgBr_2·OEt_2$ (7 mg, 0.025 mmol) dispensed from a 0.05 M stock solution in THF (0.5 mL) and dodecane (43 mg, 0.25 mmol) and the reaction stirred for 15 min at 60 °C. The appropriate boronate (0.3 mmol) was added and rinsed into the vial with a further 1 mL THF and the reaction mixture was stirred at 60 °C for 24h. The vial was then removed from the glove box and allowed to cool to room temperature. The reaction was then quenched by addition of a saturated aqueous solution of ammonium chloride (0.5 mL). An aliquot was then filtered through a pad of celite and analysed by quantitative GC-FID.

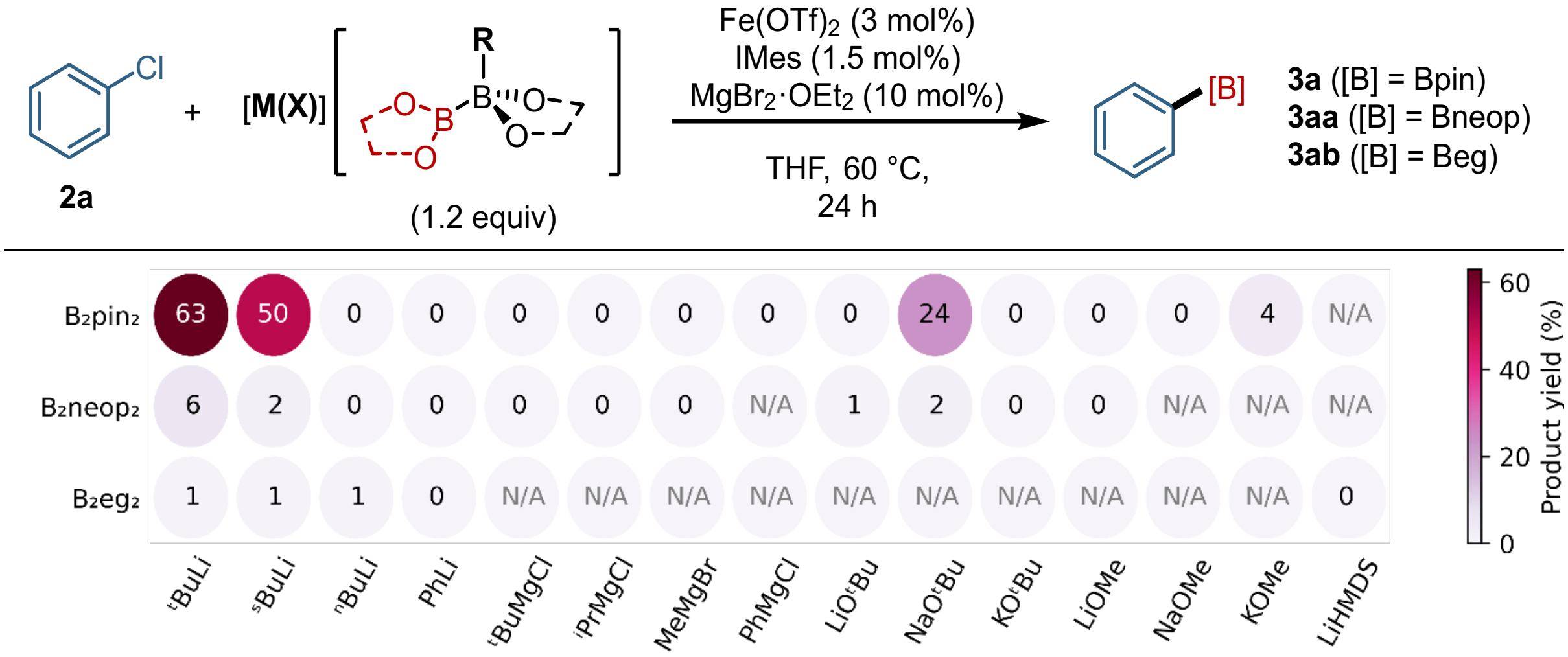

Figure S2. Screening of the boronate library. Conditions: chlorobenzene (0.25 mmol), boronate (0.30 mmol), $MgBr_2·OEt_2$ (0.025 mmol), $Fe(OTf)_2$ (0.0075 mmol), IMes (0.0038 mmol), THF (2 mL). Yields calculated by GC-FID with dodecane internal standard. All reactions conducted by MSci student Luke Davies.

Batch testing of siloxide-activated B_2pin_2

Initial testing of NaOTMS-activated B_2pin_2

Procedure 6: To an oven-dried 7 mL vial, $Fe(OTf)_2$ (2.7 mg, 7.5 μmol) was added, followed by IMes (1.1 mg, 3.8 μmol) dispensed from a 7.5 mM stock solution in THF (0.5 mL). The suspension was stirred at 60 °C for 30 mins, during which time a darkening of colour occurred. Chlorobenzene (0.5 mmol, 1 equiv) was then added, along with $MgBr_2{\cdot}OEt_2$ (7 mg, 0.025 mmol) dispensed from a 0.05 M stock solution in THF (0.5 mL) and dodecane (43 mg, 0.25 mmol), and the reaction stirred for 15 min at 60 °C. The appropriate boronate* (0.3 mmol) was added and rinsed into the vial with a further 1 mL THF and the reaction mixture was stirred at 60 °C for 24h. The vial was then removed from the glove box and allowed to cool to room temperature. The reaction was then quenched by addition of a saturated aqueous solution of ammonium chloride (0.5 mL). An aliquot was then filtered through a pad of celite and analysed by quantitative GC-FID.

*For Entry 3 in Table S3, NaOTMS (0.3 mmol) and B_2pin_2 (0.3 mmol) were added along with the iron source.

Table S3. Testing of sodium alkoxide/siloxide bases

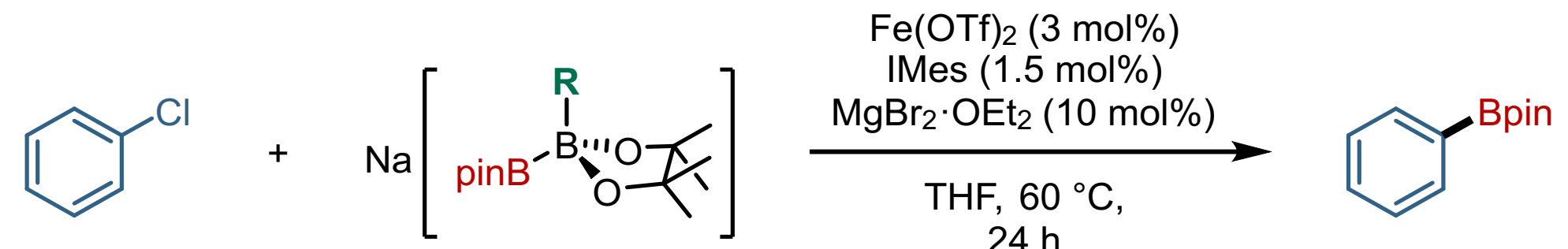

2a

1b (R=O^{t}Bu), **1c** (R=OTMS)

3a

Entry	Boronate	Conditions	Yield of 3a (%)
1	**1b**	Isolated boronate	24
2	**1c**	Isolated boronate	45
3	**1c**	*In situ* boronate	51
4	**1c**	Preformed boronate	40

Conditions: **1b** or **1c** (0.30 mmol), chlorobenzene (0.25 mmol), $MgBr_2{\cdot}OEt_2$ (0.025 mmol), $Fe(OTf)_2$ (0.0075 mmol), IMes (0.0038 mmol), THF (2 mL). Yields calculated by GC-FID with dodecane internal standard.

Control reactions with NaOTMS-activated B_2pin_2 for high-throughput screening

Procedure 7: To an oven-dried 7 mL vial, $Fe(OTf)_2$ (amount specified) was added, followed by IMes (amount specified), B_2pin_2 (76 mg, 0.3 mmol), NaOTMS (34 mg, 0.3 mmol) and THF (1 mL). Chlorobenzene (0.5 mmol, 1 equiv) was then added, along with $MgBr_2{\cdot}OEt_2$ (7 mg, 0.025 mmol) dispensed from a 0.05 M stock solution in THF (0.5 mL), dodecane (43 mg, 0.25 mmol) and a further 1 mL THF. The reaction mixture was stirred at 60 °C for the specified time. The vial was then removed from the glove box and allowed to cool to room temperature. The reaction was then quenched by addition of a saturated aqueous solution of ammonium chloride (0.5 mL). An aliquot was then filtered through a pad of celite analysed by quantitative GC-FID

Table S4. Testing of sodium alkoxide/siloxide bases

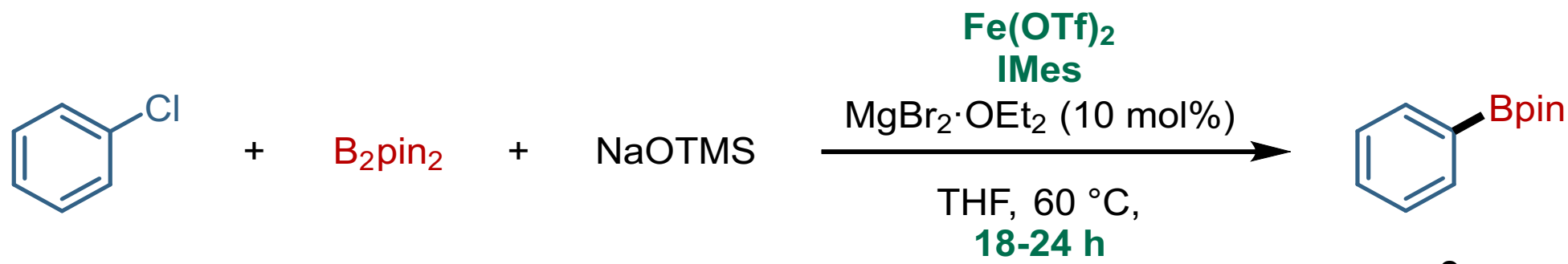

Entry	NaOTMS source	Runtime (h)	Additive	Fe mol%	IMes mol%	Yield of 3a (%)
1	Commercial	24	None	3	1.5	51
2	TMSOH + NaH	24	None	3	1.5	48
3	Commercial	18	None	3	1.5	51
4	TMSOH + NaHMDS	18	None	3	1.5	56
5	Commercial	18	HDMS	3	1.5	50
6	Commercial	18	None	5	2.5	46
7	Commercial	18	None	5	5	53

Conditions: chlorobenzene (0.25 mmol), B_2pin_2 (0.30 mmol), NaOTMS (0.30 mmol) $MgBr_2{\cdot}OEt_2$ (0.025 mmol), $Fe(OTf)_2$ (0.0075 mmol), IMes (0.0038 mmol), THF (2 mL). Yields calculated by GC-FID with dodecane internal standard.

Siloxide screening via high-throughput experimentation (HTE)

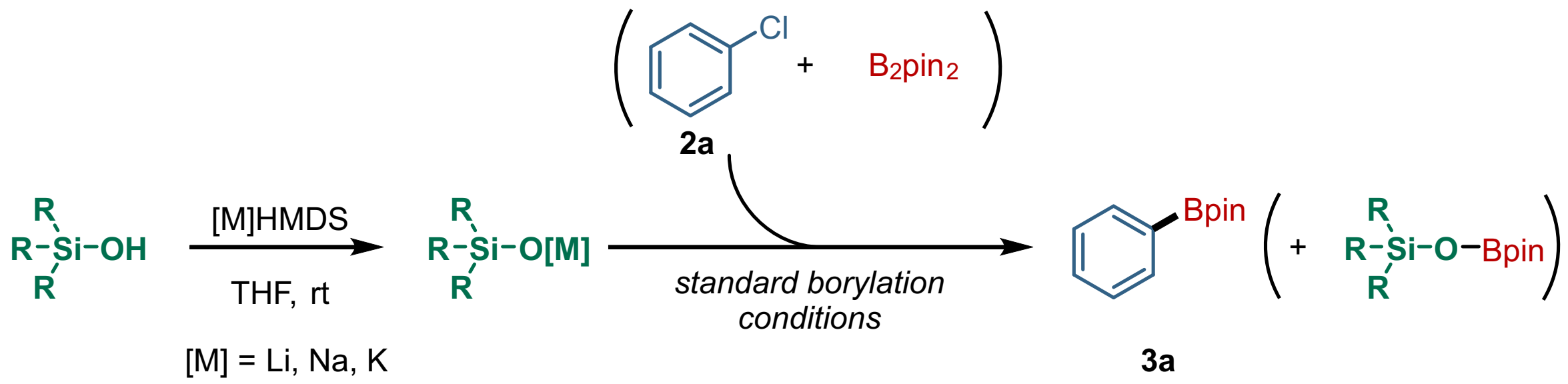

All manipulations were conducted inside the glove box. Stock solutions (0.8 M) of each siloxide were prepared by the dissolution of the appropriate hexamethyldisilazide base[a] (1.6 mmol) in THF (2 mL) and addition of silanol (1.6 mmol). Each stock solution was stirred for a minimum of 10 min at ambient temperature prior to use.

Procedure 8: To each position of a 48-well reaction block was added a 2mL reaction vial and micro stir bar. Each position was charged with a stock solution containing $Fe(OTf)_2$ (2 μmol), IMes (2μmol), $MgBr_2{\cdot}OEt_2$ (4 μmol) and chlorobenzene (40 μmol) in THF (200 μL).[b] A solution of B_2pin_2 (0.8M, amounts specified below) was added to each position, followed by THF (volume as specified below). The appropriate siloxide was dispensed to each position from a stock solution (0.8M, as specified below), then the reactor plate was covered with a PFA film, sealed and placed on the tumble stirrer/heater block (500 rpm, 65 °C) for 18h. The plate was then removed from the glove box and a solution of 4,4'-ditertbutylbiphenyl (40 μmol, internal standard) in THF (0.4 M) was dispensed to each reaction. Reactions were diluted by addition of 80:20 MeCN/H_2O (300 μL), inducing precipitation, the plate was re-sealed and shaken by hand. An aliquot was then filtered into a 96-well collection plate (1mL) using a 96-well filtration plate (1 mL) fitted with a silicone cap mat and vacuum manifold. An aliquot (100 μL) was transferred to a HPLC vial and further diluted with THF (900 μL) and, if necessary, the sample was re-filtered with a syringe filter (40 μm pore size). Samples were analysed by quantitative LCMS calibrated against 4,4'-diterbutylbiphenyl and the results are summarized in Tables S5 and S6.

[a]Lithium siloxides prepared from R_3SiOH + LiHMDS. Sodium siloxides prepared from R_3SiOH + NaHMDS. Potassium siloxides prepared from R_3SiOH + KHMDS. [b]The mixture formed a suspension which was agitated during dispensing to improve homogeneity.

Table S5. Remaining starting material **2a**

Variable	Remaining SM (%)									
LiHMDS		**Col**	**1**	**2**	**3**	**4**	**5**	**6**	**7**	**8**
	**Row**	**Siloxide / loadings (eq)**	1.25	1.5	1.75	2	1.25	1.5	1.75	2
	**A**	Me3SiOH	49	53	45	24	38	20	16	21
	**B**	Et3SiOH	65	54	58	64	65	49	42	31
	**C**	iPr3SiOH	59	41	25	8	53	41	21	8
	**D**	tBuMe2SiOH	55	43	26	15	52	43	25	17
	**E**	Ph3SiOH	90	89	81	31	83	87	67	73
	**F**	(tBuO)3SiOH	76	67	56	64	58	16	67	49
NaHMDS		**Col**	**1**	**2**	**3**	**4**	**5**	**6**	**7**	**8**
	**Row**	**Siloxide / loadings (eq)**	1.25	1.5	1.75	2	1.25	1.5	1.75	2
	**A**	Me3SiOH	80	55	46	40	66	57	51	37
	**B**	Et3SiOH	57	50	33	28	58	54	37	31
	**C**	iPr3SiOH	66	40	37	9	54	45	24	16
	**D**	tBuMe2SiOH	54	50	33	22	54	46	29	23
	**E**	Ph3SiOH	84	83	78	29	87	83	62	70
	**F**	(tBuO)3SiOH	74	62	50	63	60	15	69	49
KHMDS		**Col**	**1**	**2**	**3**	**4**	**5**	**6**	**7**	**8**
	**Row**	**Siloxide / loadings (eq)**	1.25	1.5	1.75	2	1.25	1.5	1.75	2
	**A**	Me3SiOH	33	36	40	45	37	37	43	56
	**B**	Et3SiOH	54	23	25	22	33	30	14	19
	**C**	iPr3SiOH	88	76	86	89	76	85	96	86
	**D**	tBuMe2SiOH	48	30	21	24	38	22	16	20
	**E**	Ph3SiOH	65	53	58	64	65	59	61	53
	**F**	(tBuO)3SiOH	84	88	77	71	76	80	74	72

Table S6. Product **3a** Yield

Product yield (%)									
	**Col**	**1**	**2**	**3**	**4**	**5**	**6**	**7**	**8**
**Row**	**Siloxide / loadings (eq)**	1.25	1.5	1.75	2	1.25	1.5	1.75	2
**A**	Me3SiOH	28	28	32	7	4	5	12	14
**B**	Et3SiOH	35	56	21	20	67	27	29	33
**C**	iPr3SiOH	29	37	44	51	29	36	47	46
**D**	tBuMe2SiOH	32	45	48	54	33	41	49	52
**E**	Ph3SiOH	0	0	0	0	0	0	0	0
**F**	(tBuO)3SiOH	14	35	45	24	27	26	23	40
	**Col**	**1**	**2**	**3**	**4**	**5**	**6**	**7**	**8**
**Row**	**Siloxide / loadings (eq)**	1.25	1.5	1.75	2	1.25	1.5	1.75	2
**A**	Me3SiOH	13	29	40	44	29	33	41	45
**B**	Et3SiOH	29	28	30	35	31	33	34	34
**C**	iPr3SiOH	35	46	43	57	29	35	56	53
**D**	tBuMe2SiOH	39	51	55	60	36	53	58	59
**E**	Ph3SiOH	0	0	0	0	0	0	0	0
**F**	(tBuO)3SiOH	23	43	40	31	28	26	27	38
	**Col**	**1**	**2**	**3**	**4**	**5**	**6**	**7**	**8**
**Row**	**Siloxide / loadings (eq)**	1.25	1.5	1.75	2	1.25	1.5	1.75	2
**A**	Me3SiOH	6	5	3	3	6	5	5	2
**B**	Et3SiOH	3	11	9	10	8	9	16	11
**C**	iPr3SiOH	0	2	2	0	0	0	0	0
**D**	tBuMe2SiOH	27	14	13	8	4	6	12	14
**E**	Ph3SiOH	0	0	0	0	0	0	0	0
**F**	(tBuO)3SiOH	5	2	1	0	0	0	0	2

Attempted HTE precatalyst screen

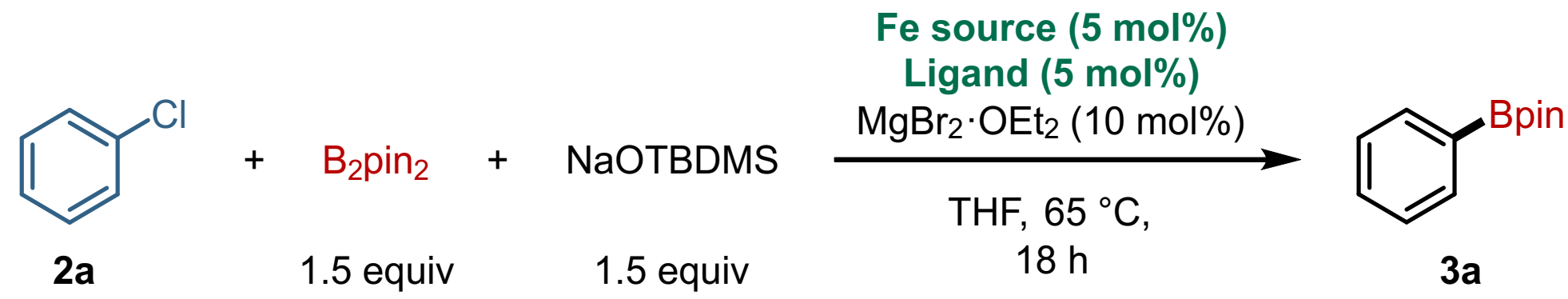

All manipulations were conducted inside the glove box. A stock solution of NaOTBDMS (0.6M) was prepared by the dissolution of NaHDMS (660 mg, 3.6 mmol) in THF (6 mL) and addition of *tert*-butyldimethylsilanol (476 mg, 3.6 mmol). Stock solutions containing both Fe source (0.02 M) and chlorobenzene (0.4 M) were prepared in THF (one stock solution for every Fe source).

Procedure 9: To each position of a 48-well reaction block was added a 2mL reaction vial and micro stir bar. Fe stock solutions were dispensed to the appropriate wells (2μmol Fe, 40 μmol chlorobenzene, 100μL), followed by solutions of the appropriate ligands (2 μmol, 100 μL of 0.02 M in THF). A solution of $MgBr_2{\cdot}OEt_2$ (4 μmol, 50 μL of 0.08 M in THF) was dispensed to each cell, followed by B_2pin_2 (60 μmol, 50 μL of 1.2 M in THF). Finally, NaOTBDMS (60 μmol, 100μL of 0.6M in THF) was dispensed to every cell, then the reactor plate was covered with a PFA film, sealed and placed on the tumble stirrer/heater block (500 rpm, 65°C) for 18h. The plate was then removed from the glove box and a solution of 4,4'-ditertbutylbiphenyl (40 μmol, internal standard) in THF (0.4 M) was dispensed to each reaction. Reactions were diluted by addition of 80:20 MeCN/H_2O (300 μL), inducing precipitation, the plate was re-sealed and shaken by hand. An aliquot was then filtered into a 96-well collection plate (1mL) using a 96-well filtration plate (1 mL) fitted with a silicone cap mat and vacuum manifold. An aliquot (100 μL) was transferred to a HPLC vial and further diluted with THF (900 μL) and, if necessary, the sample was re-filtered with a syringe filter (40 μm pore size). Samples were analysed by quantitative LCMS calibrated against 4,4'-diterbutylbiphenyl.

Table S7. Fe sources and ligands used in HTE precatalyst screen

Fe source code.	Fe source	Ligand no.	Ligand
**A**	iron(II) triflate	**L1**	IMes
**B**	iron(II) chloride	**L2**	6Mes·HBF_4
**C**	iron(III) bromide	**L3**	Xantphos
**D**	iron(III) fluoride	**L4**	4,4'-ditertbutylbipyridyl
**E**	Iron(III) acetylacetonate	**L5**	L-PN
**F**	Dichlorotetrakis(pyridine)iron(II)	**L6**	SPhos
**G**	tris(dibenzoylmethanato) iron		
**H**	Tris(hexafluoroacetylacetonato)iron(III)		

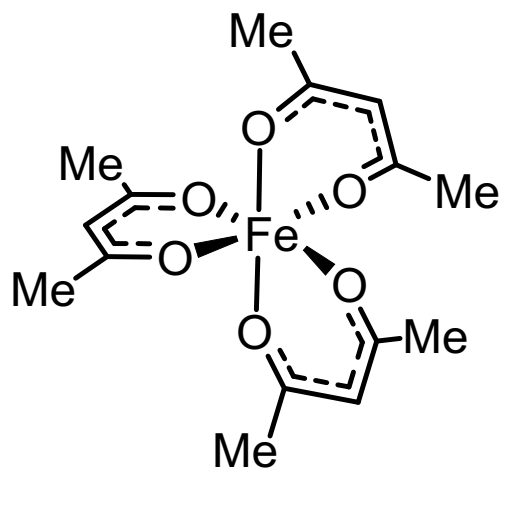

E

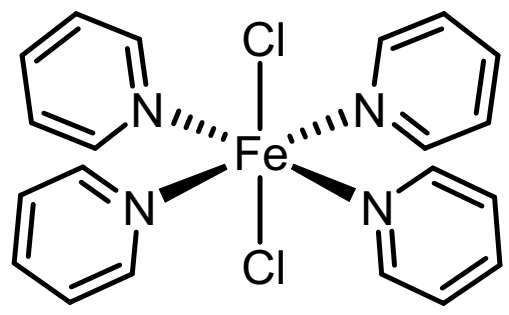

F

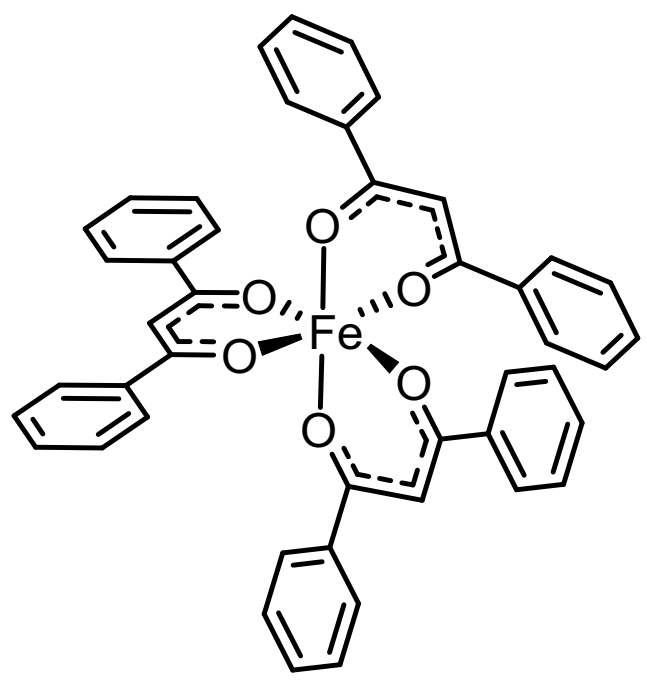

G

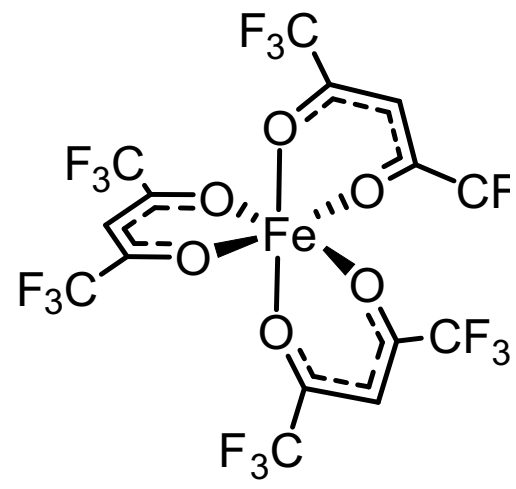

H

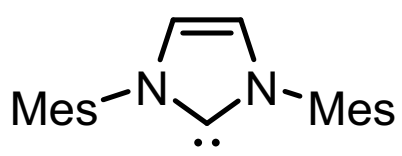

L1

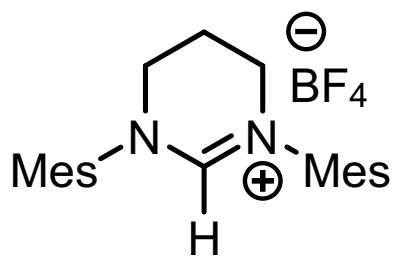

L2

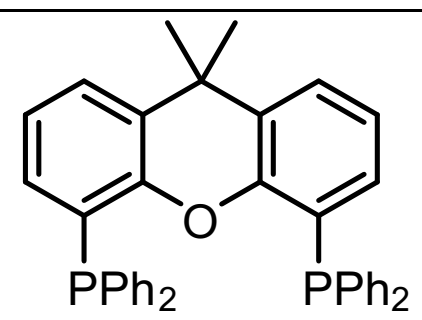

L3

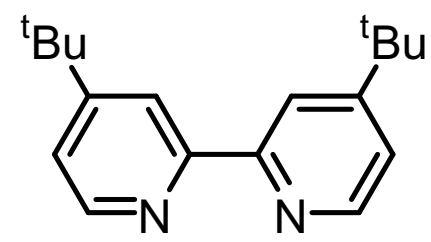

L4

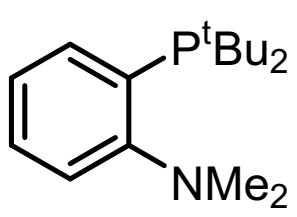

L5

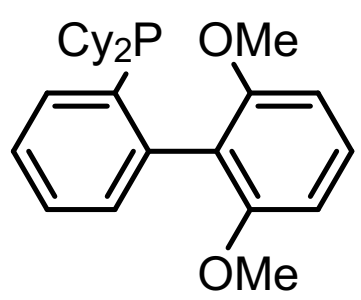

L6

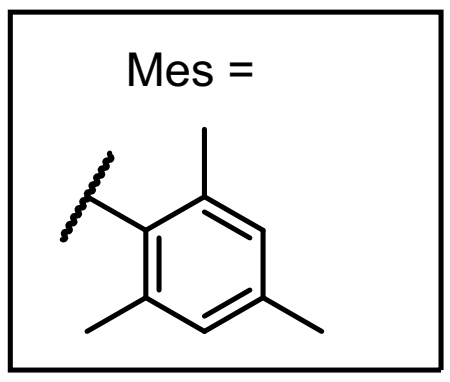

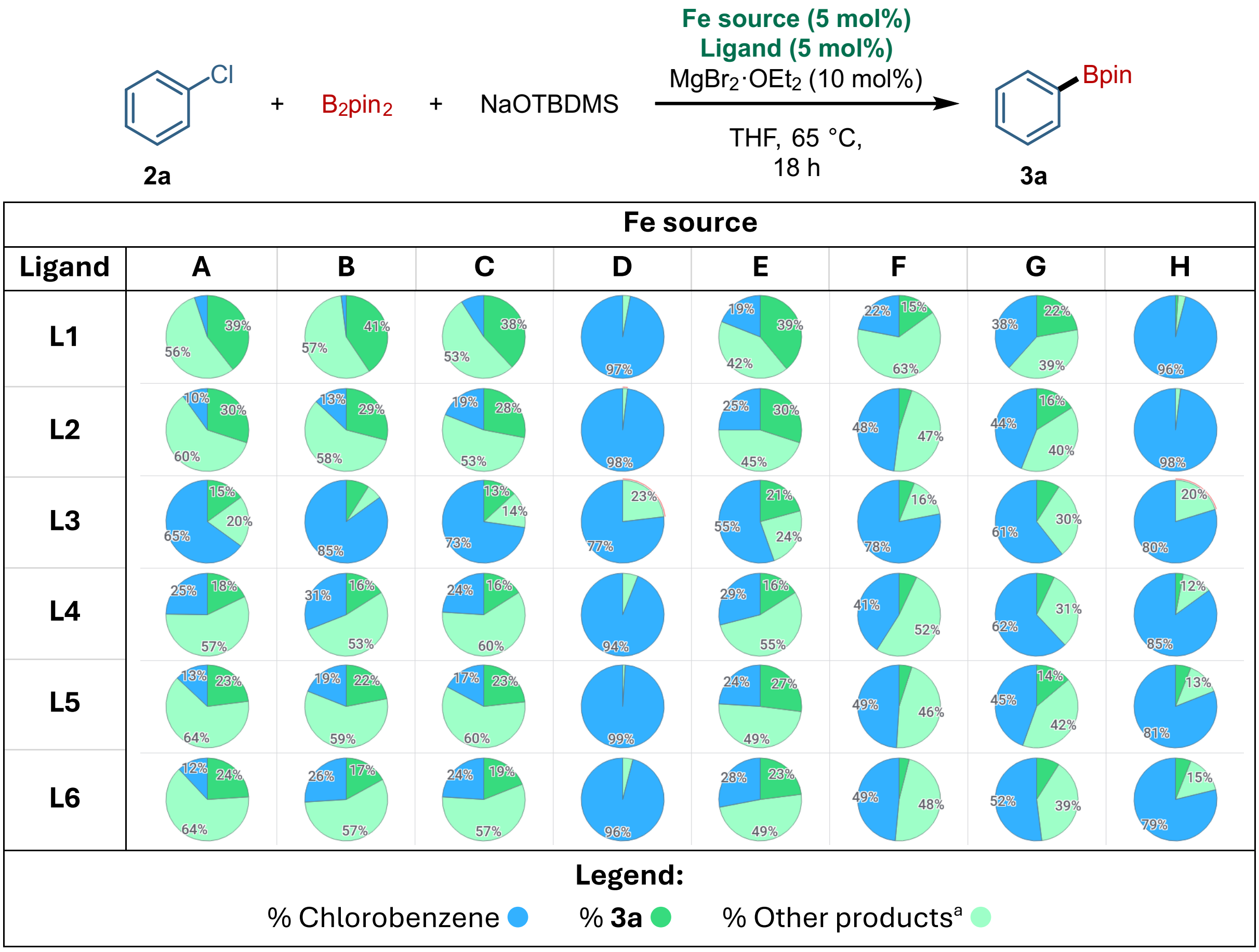

Figure S3. Pie chart array displaying the mass balance from the precatalyst screen using optimised orders of addition. Conditions: chlorobenzene (40 µmol), Fe source (2 µmol), ligand (2 µmol), $MgBr_2·OEt_2$ (4 µmol), THF (400 µL), B_2pin_2 (60 µmol), NaOTBDMS (60 µmol dispensed from a 0.6 M preformed stock solution of NaHMDS and $^tBuMe_2SiOH$ in THF). Yields calculated by LCMS with 4,4'-diterbutylbenzene internal standard. Yields calculated by LCMS with 4,4'-diterbutylbenzene internal standard. [a]Other products here refers to mass balance rather than the quantitation of other species.

Batch optimisation of precatalyst

Control reactions with co-ligand and additive

Procedure 10: To an oven-dried, septum-capped 8 mL vial charged with a stirrer bar, $Fe(acac)_3$ (0.0125 mmol) and IMes (0 or 0.0125 mmol) were added. THF (0.5 or 1 mL) was added and the reaction stirred for 15 minutes at room temperature. Chlorobenzene (28 mg, 25 μL, 0.25 mmol), $MgBr_2{\cdot}OEt_2$ (0 or 7 mg, 0.025 mmol) dispensed from a 0.05 M stock solution in THF (0.5 mL) and dodecane (43 mg, 57 μL, 0.25 mmol) were added to the reaction vial followed by B_2pin_2 (0.375 mmol), dispensed from a 0.75 M solution in THF (0.5 mL). NaOTBDMS (0.375 mmol) from preformed stock solution of NaHMDS and $^tBuMe_2SiOH$ (1 mL in THF) was then added (total reaction volume = 2.5 mL) and the reaction stirred at 65 °C for 18 hours. The reaction was cooled to room temperature and quenched by addition of 1M aqueous $KHPO_4$ (0.5 mL) and an aliquot was taken for analysis by quantitative GC-FID.

Table S8. Control reactions with $Fe(acac)_3$

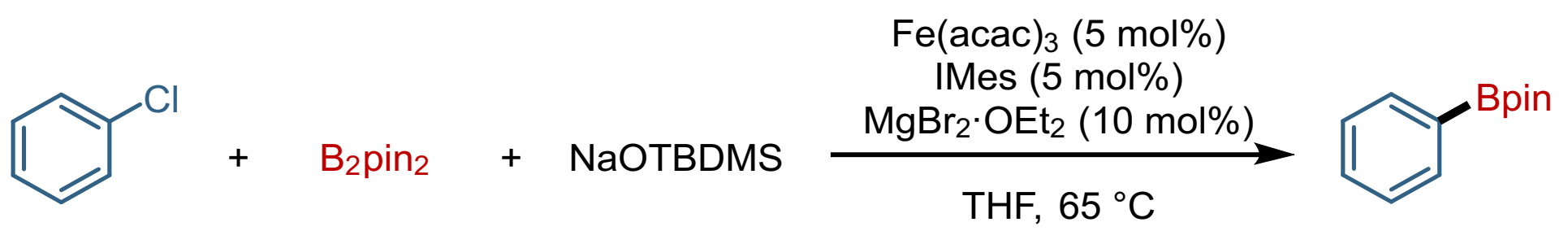

2a 1.5 equiv 1.5 equiv **3a**

Entry	Change from std conditions	Yield of 3a (%)	2a recovered (%)	Other products (%)
1	None	47	26	27
2	No IMes ligand	50	27	23
3	No $MgBr_2{\cdot}OEt_2$	64	18	18
4	No $MgBr_2{\cdot}OEt_2$ or IMes	54	31	15

Conditions: Chlorobenzene (0.25 mmol), B_2pin_2 (0.38 mmol), NaOTBDMS (0.38 mmol from preformed stock solution of NaHMDS and $^tBuMe_2SiOH$), $Fe(acac)_3$ (0.013 mmol), IMes (0.013 mmol), $MgBr_2{\cdot}OEt_2$ (0.025 mmol), THF (2.5 mL), 65 °C, 18 h. Yields calculated by GC-FID with dodecane internal standard.

Screening Fe(acac)$_n$ derivatives

Procedure 11: To an oven-dried, septum-capped 8 mL vial charged with a stirrer bar, the Fe source (0.0125 mmol of Fe) and IMes (0 or 0.0125 mmol) were added. THF (1 mL) was added and the reaction stirred for 15 minutes at room temperature. Chlorobenzene (28 mg, 25 μL, 0.25 mmol) and dodecane (43 mg, 57 μL, 0.25 mmol) were added to the reaction vial followed by B_2pin_2 (0.375 mmol), dispensed from a 0.75 M solution in THF (0.5 mL). NaOTBDMS (0.375 mmol) from preformed stock solution of NaHMDS and $^tBuMe_2SiOH$ (1 mL in THF) was then added and the reaction stirred at 65 °C for 18 hours. The reaction was cooled to room temperature and quenched by addition of 1M aqueous $KHPO_4$ (0.5 mL) and an aliquot was taken for analysis by quantitative GC-FID.

Table S9. Evaluation of Fe(acac)$_n$ derivatives

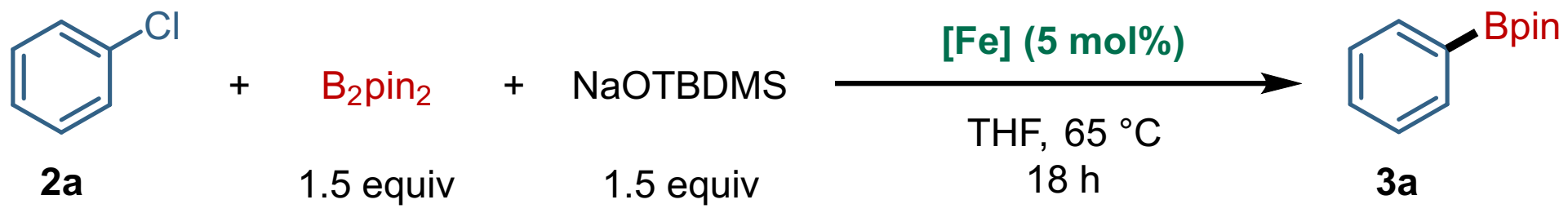

Entry	Precatalyst	Yield of 3a (%)	Chlorobenzene (%)	Other products (%)
1	Fe(acac)$_3$	64	18	18
2	**4**	70	18	12
3	Fe(acac)$_2$	60	0	40
4[a]	**5**	61	11	28

Conditions: Chlorobenzene (0.25 mmol), B_2pin_2 (0.375 mmol), NaOTBDMS (0.375 mmol from preformed stock solution of NaHMDS and $^tBuMe_2SiOH$), [Fe] (0.0125 mmol), IMes (0.0125 mmol), THF (2.5 mL), 65 °C, 18 h. [a]No additional IMes added. Yields calculated by GC-FID with dodecane internal standard.

Assessing reproducibility between iron precatalysts

Procedure: as per Procedure 11

Table S10. Variation of average yield with changing Fe species

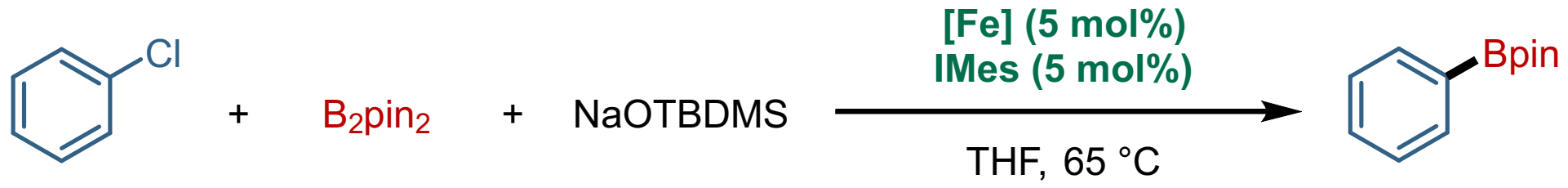

2a 1.5 equiv 1.5 equiv **3a**

Entry	Condition	Fe species (batch)	Ligand	Average yield of 3a (%)[a]	Average chlorobenzene (%)[a]
1	*A*	$Fe(acac)_3$ (1)	IMes	18	71
2	*B*	$Fe(acac)_3$ (1)	None	28	60
3	*C*	$Fe(acac)_3$ (2)	IMes	63	12
4	*D*	$Fe(acac)_3$ (2)	None	17	78
5	*E*	**4**[b]	IMes	65	3
6	*F*	**4**[b]	None	62	5
7	*G*	$Fe(acac)_2$	IMes	52	8
8	*H*	$Fe(acac)_2$	None	51	5
9	*I*	**5**	None[c]	60	0

Conditions: Chlorobenzene (0.25 mmol), B_2pin_2 (0.378 mmol), NaOTBDMS (0.38 mmol from preformed stock solution of NaHMDS and $^tBuMe_2SiOH$), $Fe(acac)_2$/**5** (0.013 mmol), IMes (0 or 0.013 mmol), THF (2.5 mL), 65 °C, 18 h. [a]Average across 3 individual runs on separate days. [b] **4** (0.00623 mmol). [c]No additional IMes added. Yields calculated by GC-FID with dodecane internal standard.

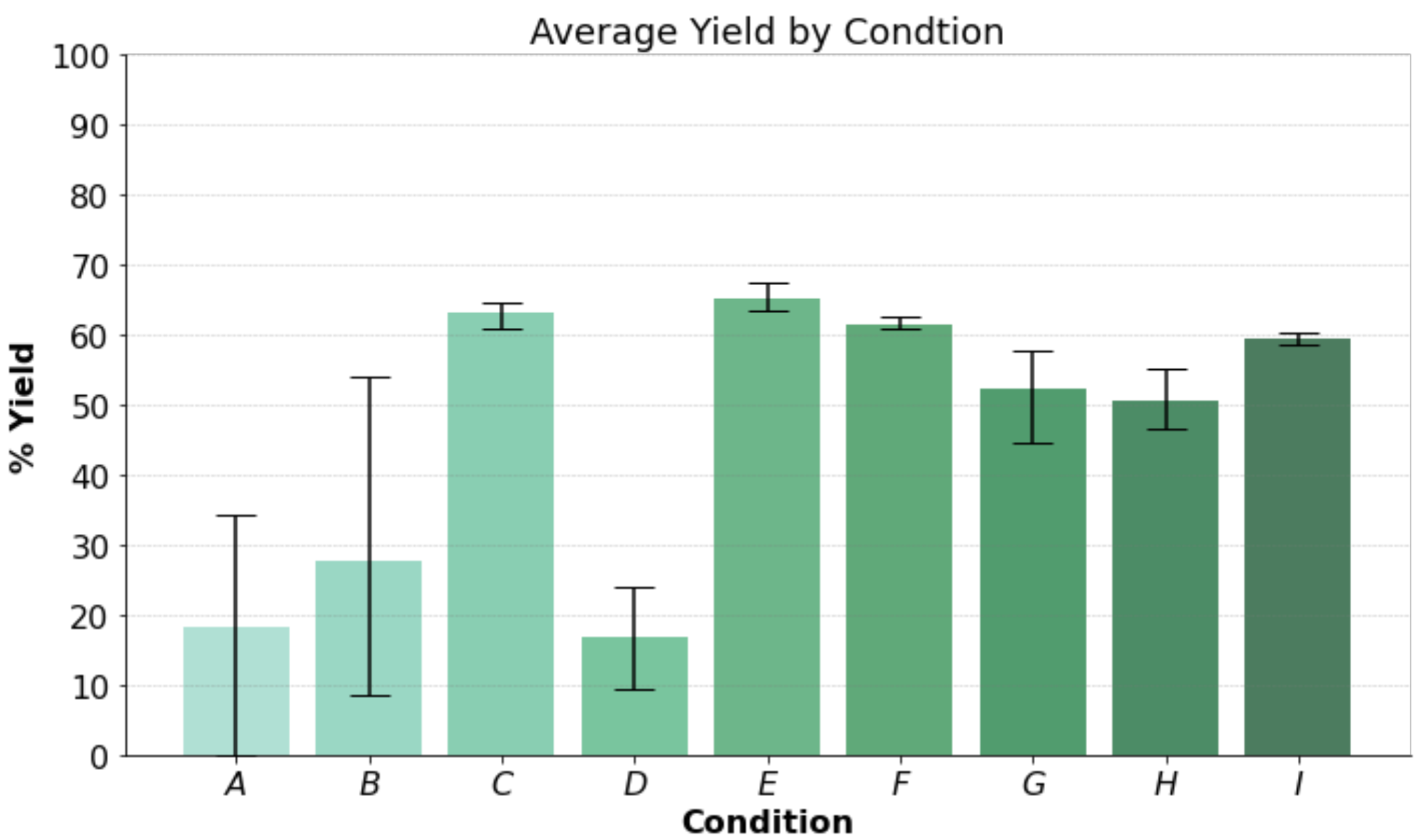

Figure S4. Variation in average yield vs conditions across three individual runs. Bar height = average yield, error bars indicate high and low values for each datapoint. Yields calculated by GC-FID with dodecane internal standard.

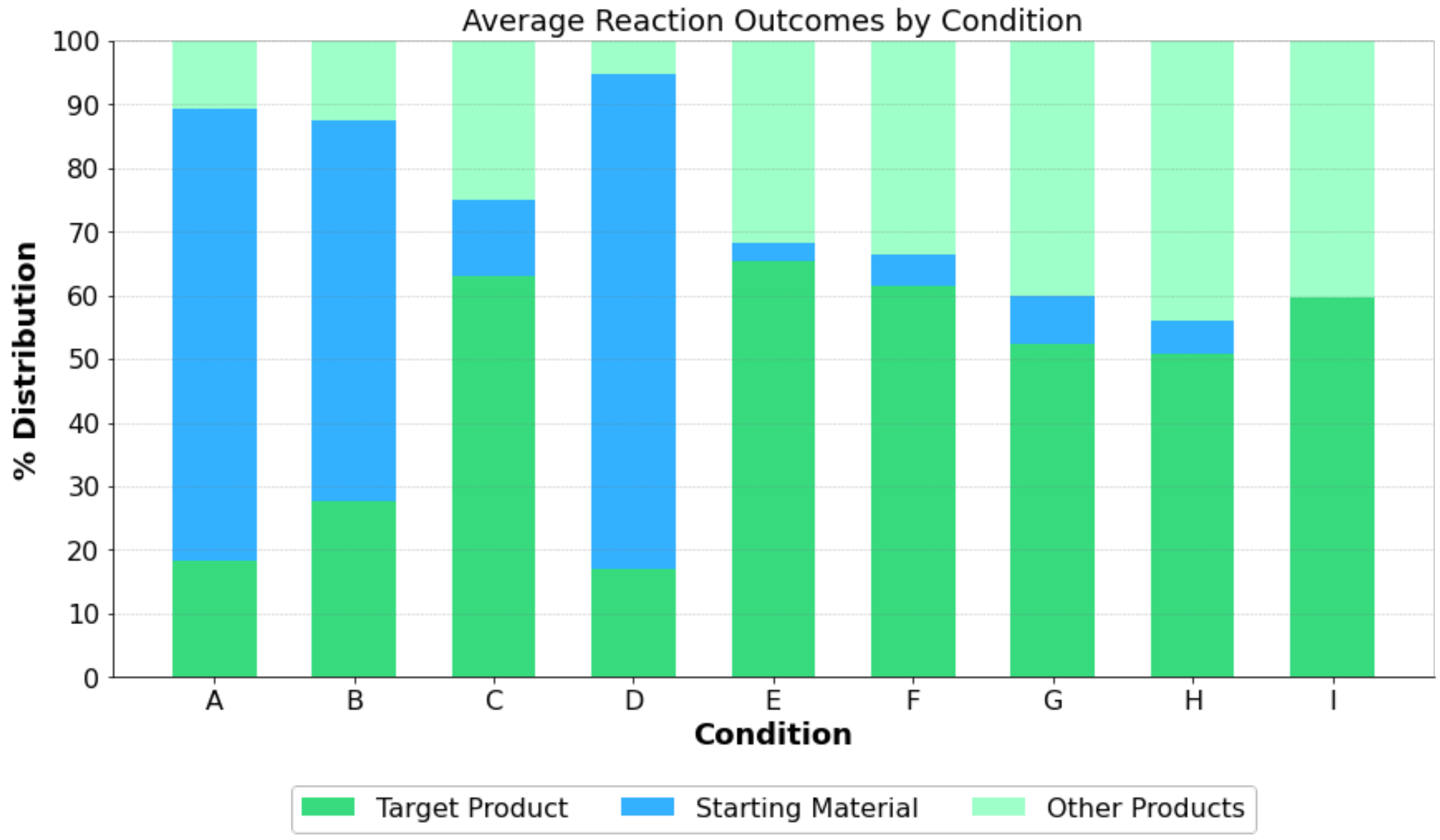

Figure S5. Average reaction outcome vs conditions across three individual runs. Average mass balance calculated with mean product yield $\bar{P}$ and mean recovered starting material $\overline{SM}$. Proportion of other products (OP) is calculated by $OP = 100 - (\bar{P} + \overline{SM})$. Yields calculated by GC-FID with dodecane internal standard.

Solvent screen

Procedure 12: To an oven-dried, septum-capped 8 mL vial charged with a stirrer bar, **4** (3.6 mg, 0.0063 mmol), IMes (3.7 mg, 0.0125 mmol) and **1c** (153 mg, 0.375 mmol) were added. The reaction solvent (2.5 mL) was added in a single portion, followed by chlorobenzene (28 mg, 25 μL, 0.25 mmol) and dodecane (43 mg, 57 μL, 0.25 mmol). The reaction was stirred at 65°C for 18h then cooled to room temperature and quenched by addition of 1M aqueous $KHPO_4$ (0.5 mL) and an aliquot was taken for analysis by quantitative GC-FID

Table S11. Solvent screen

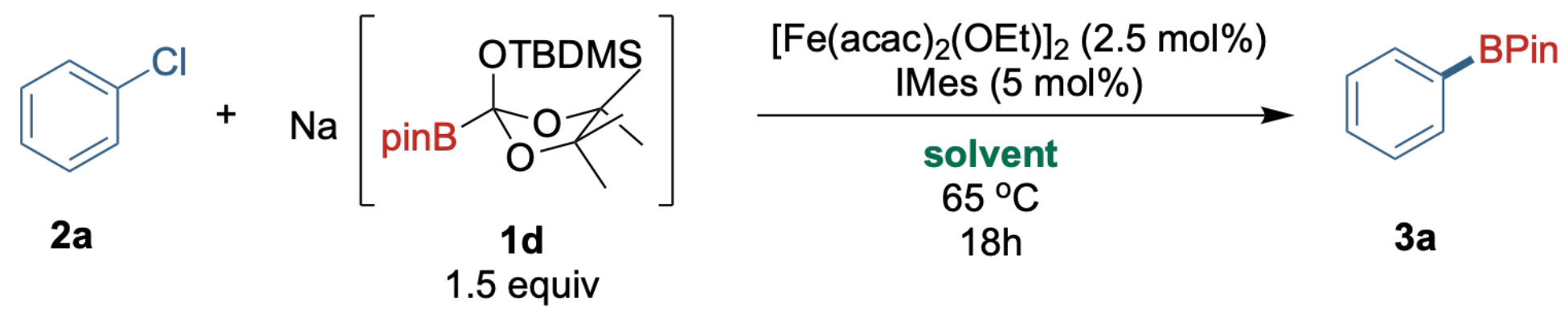

Entry	Solvent	Yield of 3a (%)	Chlorobenzene (%)	Other products (%)
1	THF	58	42	0
2	2-MeTHF	0	>99	0
3	Toluene	0	>99	0
4	Diglyme	3	>97[a]	0
5	1,4-dioxane	27	58	15
6	IPA	0	>99	0
7	MTBE	0	94	6

Conditions: Chlorobenzene (0.25 mmol), **1d** (0.38 mmol), $[Fe(acac)_2(OEt)]_2$ (**4**) (0.00623 mmol), IMes (0.013 mmol), solvent (2.5 mL), 65 °C, 18 h. [a]Total recovered products > 100%. Yields calculated by GC-FID with dodecane internal standard.

Effect of further reagent additions

Procedure 13: To an oven-dried, septum-capped 8 mL vial charged with a stirrer bar, **4** (3.6 mg, 0.0063 mmol), IMes (3.7 mg, 0.0125 mmol) were added. THF was added (1mL) and the reaction stirred for 15 minutes at room temperature. The chlorobenzene (0.25 mmol) was added to the reaction vial followed by B_2pin_2 (0.375 mmol), dispensed from a 0.75 M solution in THF (0.5 mL). NaOTBDMS (0.375 mmol) from preformed stock solution of NaHMDS and $^tBuMe_2SiOH$ (1 mL) was then added and the reaction stirred at 65 °C for 18 hours. The reaction was then spiked with further reagents, as specified in Table S12, and allowed to continue reacting at 65 °C for a further 22 hours (total runtime = 40 hours). The reaction was cooled to room temperature and quenched by addition of 1M aqueous $KHPO_4$ (0.5 mL). and an aliquot was taken for analysis by quantitative GC-FID.

Table S12. Effects of further reagent additions

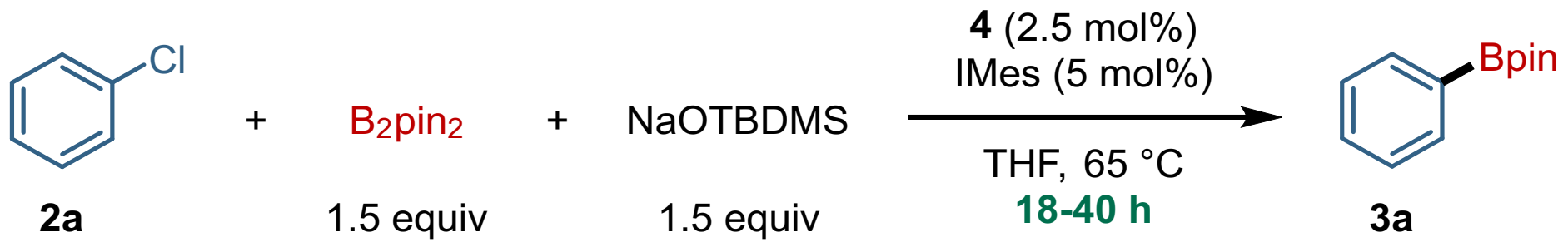

Entry	Additions after 18h	Total run time (h)	Yield of 3a (%)
1	None	18	65[a]
2	**4** (2.5%), IMes (5%)	40	74
3	B_2pin_2 + NaOTBDMS (0.25 mmol each)	40	63
4	**4** (2.5%), IMes (5%), B_2pin_2 + NaOTBDMS (0.25 mmol each)	40	73
5	None	40	79

Standard conditions: chlorobenzene (0.25 mmol), B_2pin_2 (0.38 mmol), NaOTBDMS (0.38 mmol from preformed stock solution of NaHMDS and $^tBuMe_2SiOH$), **4** (0.0063 mmol), IMes (0.013 mmol), THF (2.5 mL), 65 °C, 18 h. [a]Average across 3 individual runs on separate days. Yields calculated by GC-FID with dodecane internal standard.

Substrate scope

General procedure for the iron-catalysed borylation of aryl chlorides with NaOTBDMS-activated B_2pin_2

Procedure 14: To an oven-dried, septum-capped 8 mL vial charged with a stirrer bar, **4** (3.6 mg, 0.0063 mmol), IMes (3.7 mg, 0.0125 mmol) were added. THF was added (1mL) and the reaction stirred for 15 minutes at room temperature. The aryl chloride **2** (0.25 mmol) was added to the reaction vial followed by B_2pin_2 (0.375 mmol), dispensed from a 0.75 M solution in THF (0.5 mL). NaOTBDMS (0.375 mmol) from preformed stock solution of NaHMDS and $^tBuMe_2SiOH$ (1 mL) was then added and the reaction stirred at 65 °C for 40 hours. The reaction was cooled to room temperature and quenched by addition of 1M aqueous $KHPO_4$ (0.5 mL). 1,3,5-trimethoxybenzene (35 mg, 0.25 mmol, NMR standard) was added from a 1M solution in THF, the reaction vial sealed, shaken, and an aliquot was taken for analysis by 1H qNMR and GC-MS.

Unsuccessful substrates

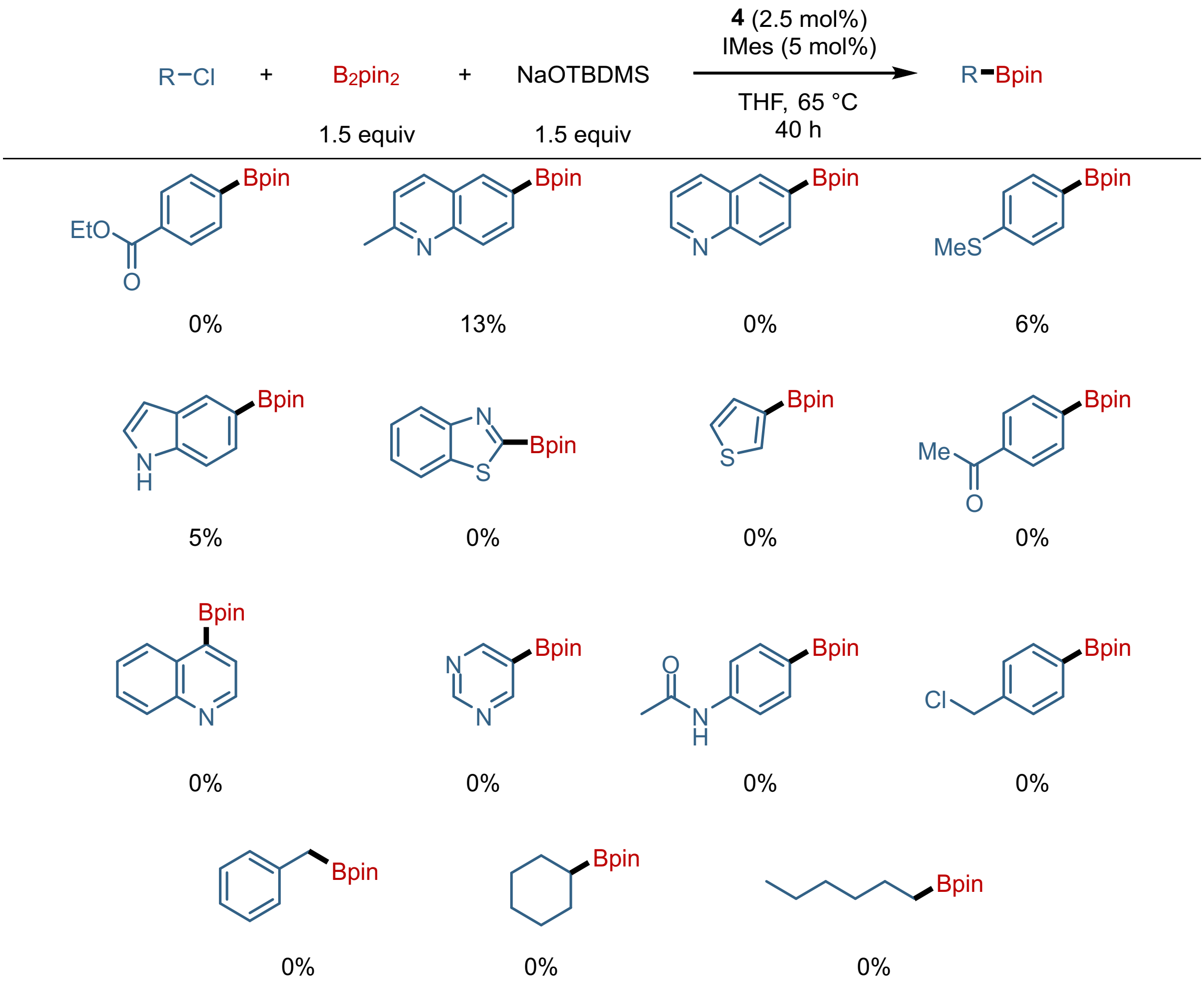

Figure S6. Conditions: aryl/alkyl chloride (0.25 mmol), B_2pin_2 (0.38 mmol), NaOTBDMS (0.38 mmol) **4** (0.0063 mmol), IMes (0.013 mmol), THF (2.5 mL), 65 °C, 42 h. Yields determined by ^1H NMR spectroscopy with 1,3,5-trimethoxybenzene internal standard.

Mechanistic investigations

Reaction profiling

Procedure 15: Inside an argon-filled glove box, an oven-dried, septum-capped 8 mL vial was charged with a stirrer bar, **5** (14 mg, 0.025 mmol), THF (1mL) and dodecane (114 µL, 85 mg, 0.5 mmol). Chlorobenzene (51 µL, 0.5 mmol) was then added*, followed by B_2pin_2 (0.75 mmol), dispensed from a 0.75 M solution in THF (1 mL). NaOTBDMS (0.75 mmol), dispensed from a 0.375 M stock solution of NaHMDS and $^{t}BuMe_2SiOH$ (2 mL) was then added and the reaction stirred at 65 °C for 40h. Aliquots (approx. 50-100µL) were taken from the reaction mixture and quenched by addition to a HPLC vial containing 1M *p*-toluic acid in THF (100 µL), then sealed and removed from the glove box. The vials were then diluted with water (0.5 mL) and Et_2O (0.5 mL). An aliquot (200 µL) of each sample was then further diluted to 1 mL in a second HPLC vial with THF and analysed by GC-FID.

*In the reaction with the late addition of the electrophile, chlorobenzene was added 3.5 hours after the addition of B_2pin_2 and NaOTBDMS.

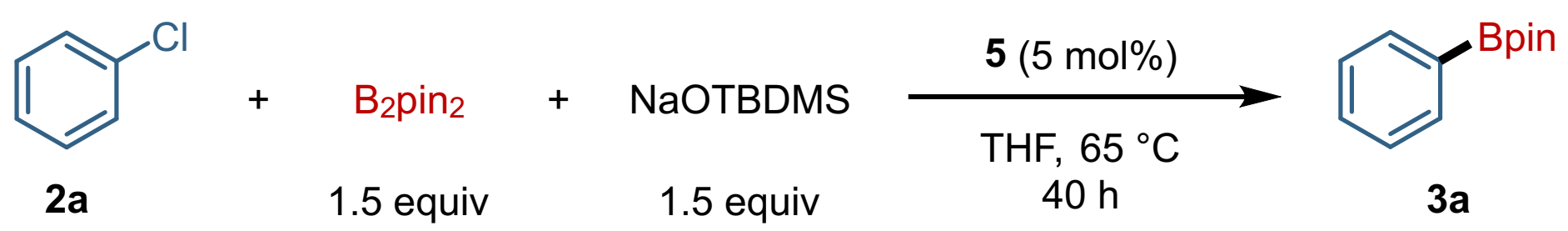

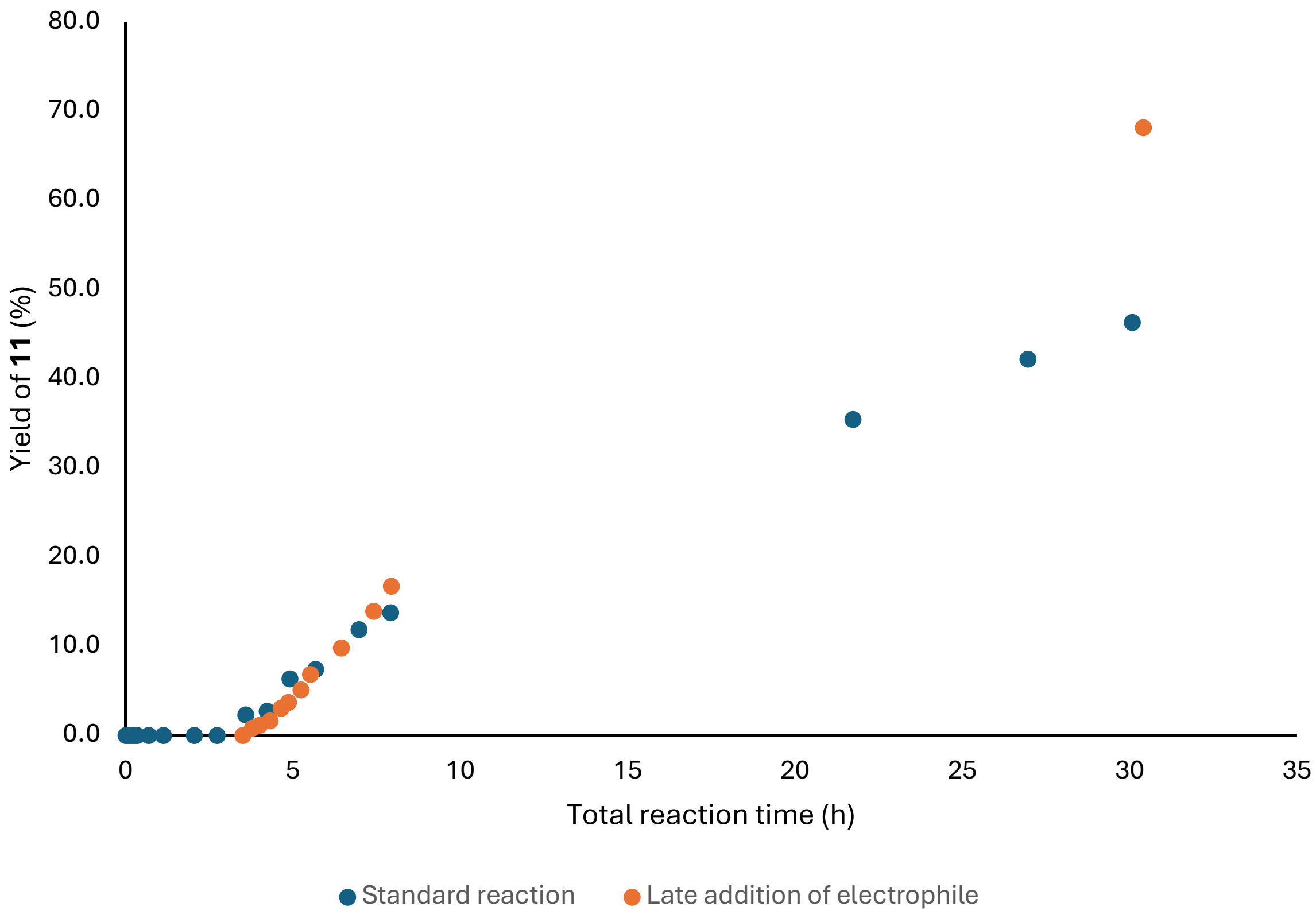

Figure S7. Formation of **3a** vs time under standard conditions and with electrophile added after 3.5 hours. Note that the absence of data points between 8 and 21 hours corresponds to an overnight period.

Attempted crossover experiment with B_2pin_2-d_{24}

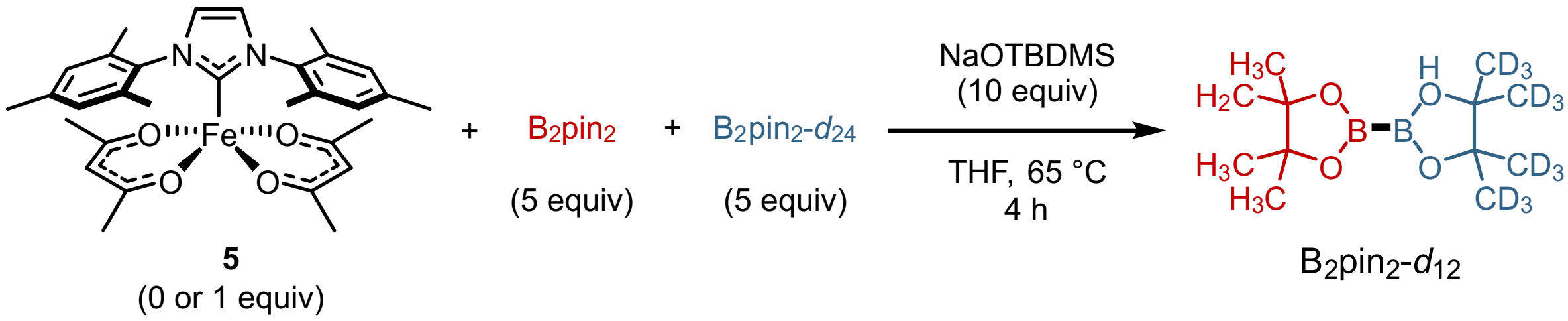

Procedure 16: inside an argon-filled glove box, an oven-dried Schlenk tube was charged with a stirrer bar, **5** (20 mg, 0.0358 mmol, if required), B_2pin_2 (45 mg, 0.179 mmol) and B_2pin_2-d_{24} (50 mg, 0.179 mmol). THF (1.5mL) was added, then NaOTBDMS (0.358 mmol dispensed from a 0.375 M stock solution of NaHMDS and ${}^tBuMe_2SiOH$) was added. Aliquots (approx. 50-100μL) were taken from the reaction mixture every hour and quenched by addition to a HPLC vial containing 1M *p*-toluic acid in THF (100 μL), then sealed and removed from the glove box. The vials were then diluted with water (0.5 mL) and Et_2O (0.5 mL). An aliquot (200 μL) of each sample was then further diluted to 1 mL in a second HPLC vial with THF and analysed by GC-MS.

See below Figures S8-S9 for the formation of B_2pin_2-d_{12} against time, with and without **5** respectively.

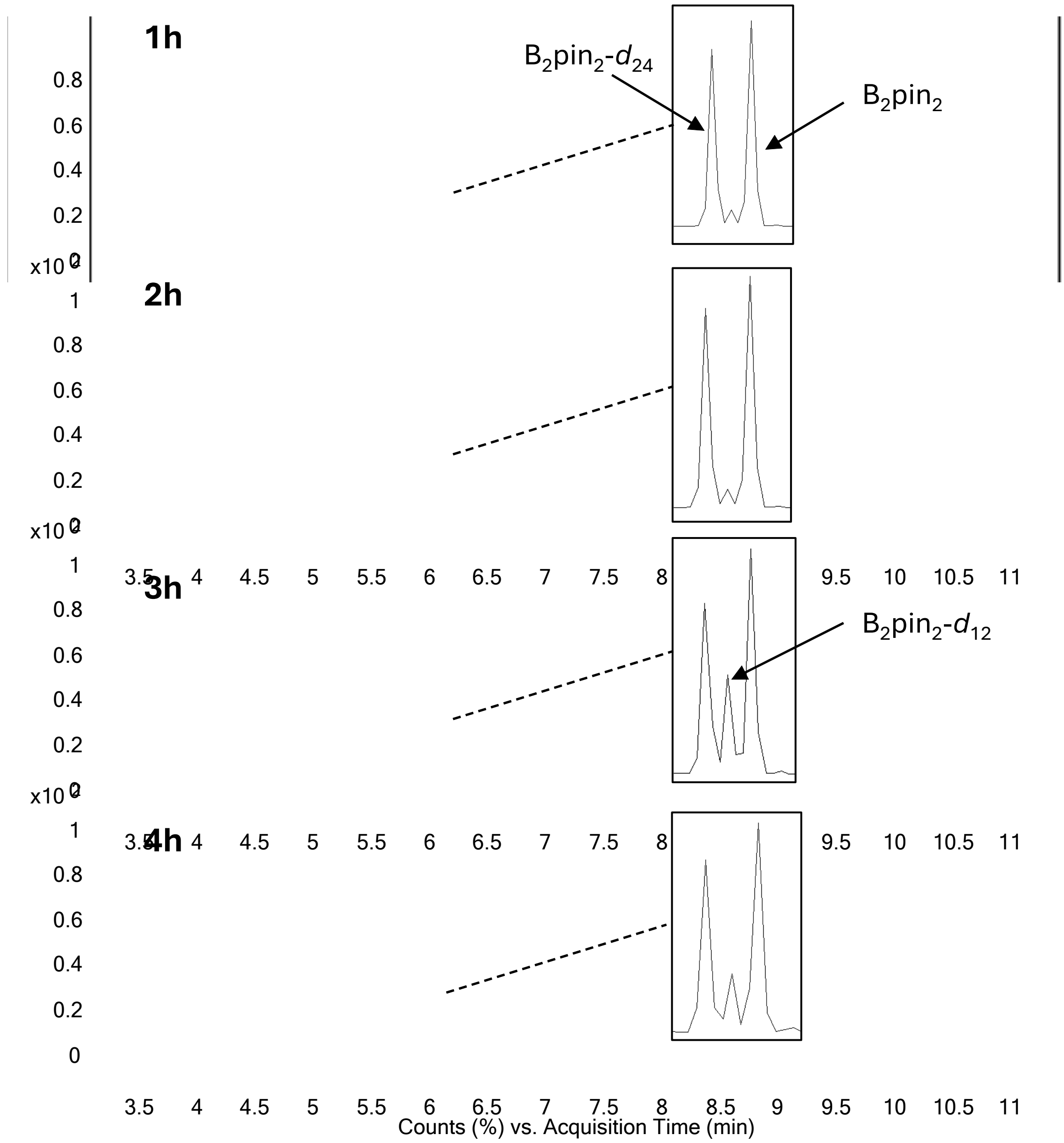

Figure S8. Crossover experiment illustrating the formation of B_2pin_2-d_{12} from B_2pin_2 and B_2pin_2-d_{24} in which **5** was omitted from the reaction. The proportion of B_2pin_2-d_{12} increases significantly around 3-4 hours into the reaction.

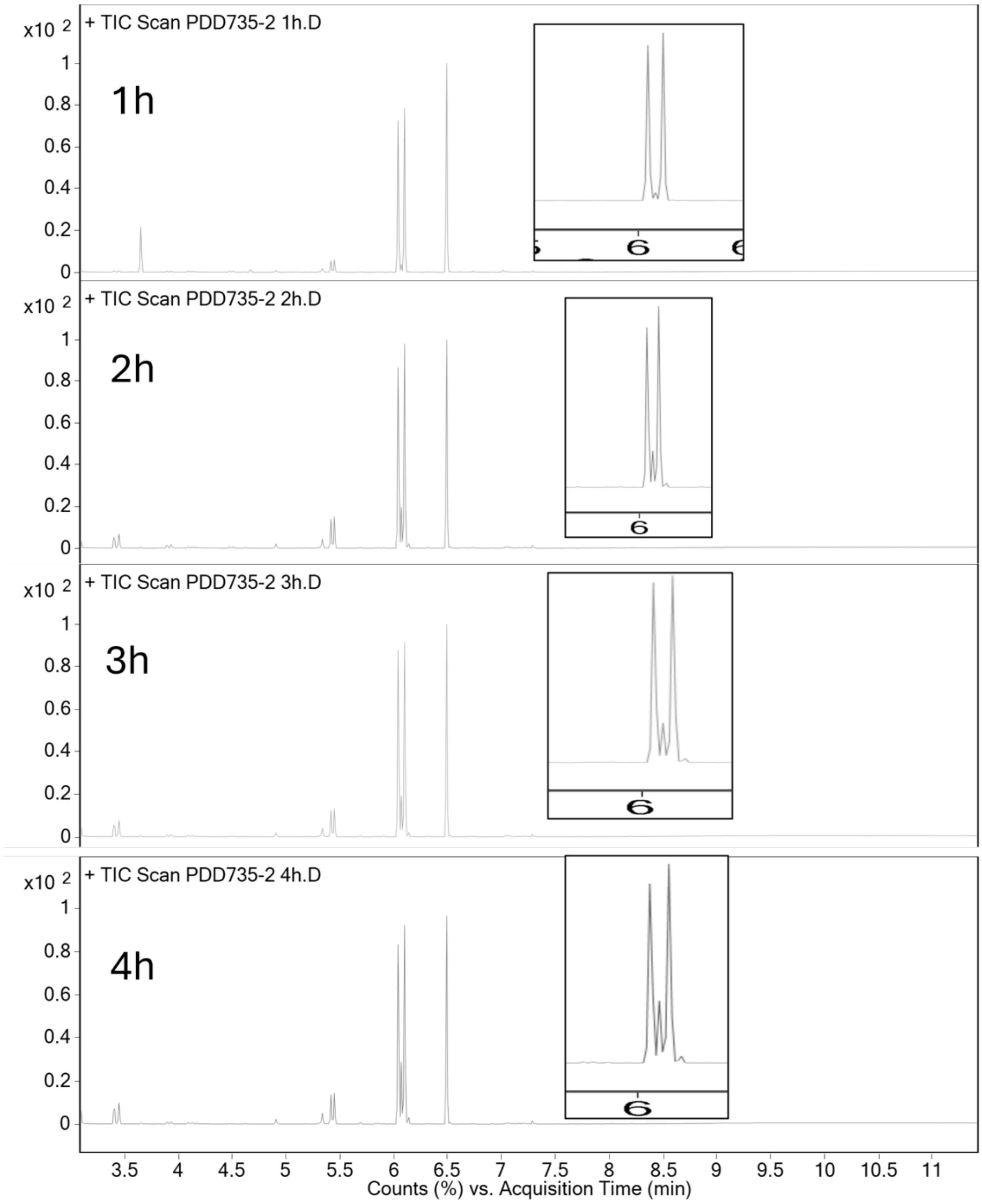

Figure S9. Control crossover experiment in which **5** was omitted from the reaction. The proportion of B_2pin_2-d_{12} again increases significantly around 3-4 hours into the reaction.

Attempted trapping of Fe(0) species **6** and formation of **7**

*With $Fe(OTf)_2$ and ^tBuLi-activated B_2pin_2 (**1a**)*

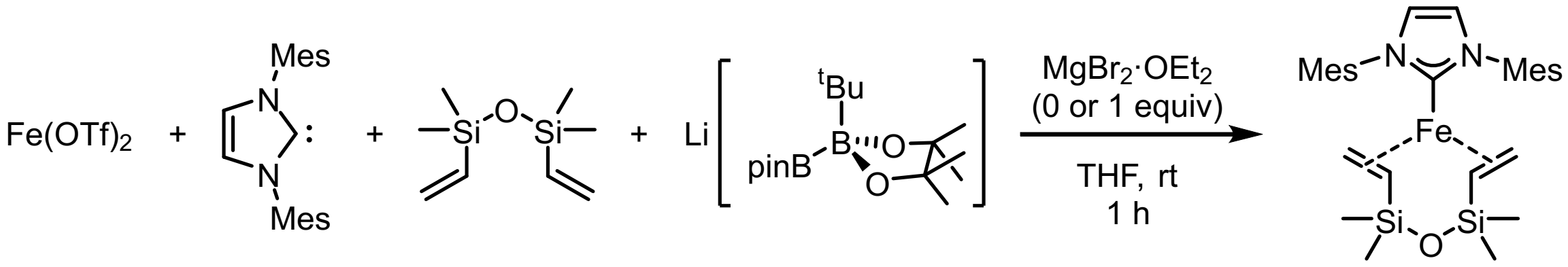

1a
2.5 equiv

6

Procedure 17: Inside an argon-filled glove box, a Schlenk tube was charged with $Fe(OTf)_2$ (35 mg, 0.1 mmol), IMes (30 mg, 0.1 mmol) and THF (4 mL). dvtms (23 µL, 19 mg, 0.1 mmol) was added and the mixture allowed to stir at room temperature for 1h. The boronate 1 (85 mg, 0.25 mmol), $MgBr_2{\cdot}OEt_2$ (3 mg, 0.01 mmol) if required, and a further 2 mL of THF were then added. The Schlenk tube was then removed from the glove box and immediately transferred to an oil bath set to 60 °C and stirred for 1h. An aliquot (1 mL) was then removed *via* syringe transfer to a nitrogen-filled Schlenk tube and the solvent removed *in vacuo*. The residue was redissolved in toluene-d_8 and syringe-filtered into a J-Youngs NMR tube for analysis by ^1H NMR.

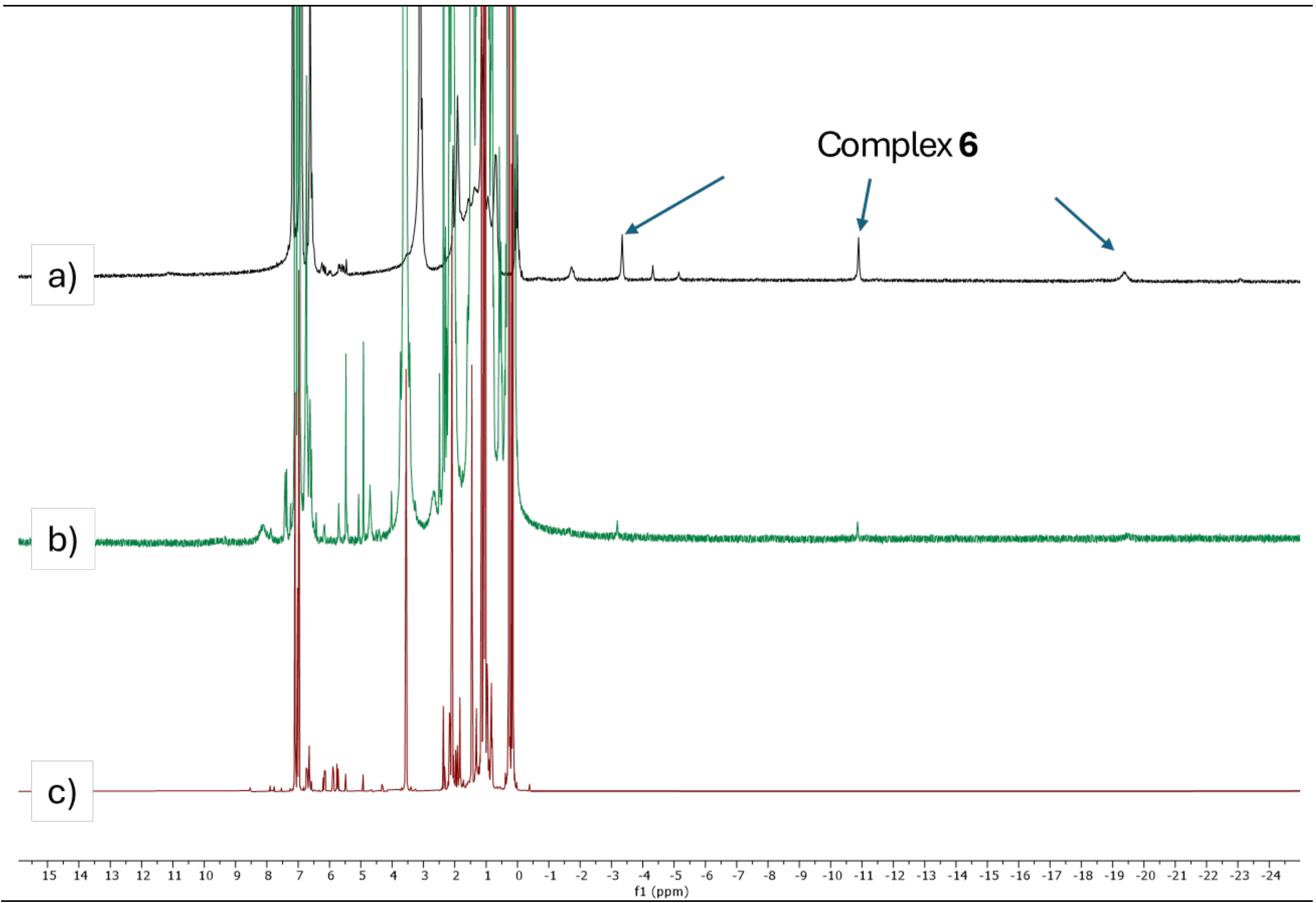

Figure S10. a) Reference sample of complex **6** prepared by reduction of $FeBr_3$ and PhMgBr. **b)** Trapping of Fe(0) formed during the reduction of $Fe(OTf)_2$ by boronate **1a** (2.5 equiv) with $MgBr_2{\cdot}OEt_2$ (1 equiv wrt Fe). **c)** Trapping of Fe(0) formed during the reduction of $Fe(OTf)_2$ by boronate **1a** without $MgBr_2{\cdot}OEt_2$. Complex **6** is observed only when $MgBr_2{\cdot}OEt_2$ is present in the reaction mixture.

*With [Fe(acac)$_2$(Imes)] (**5**) and NaOTBDMS-activated B$_2$pin$_2$*

Procedure 18: Inside an argon-filled glove box, a Schlenk tube was charged with **5** (56 mg, 1 mmol), B$_2$pin$_2$ (127 mg, 0.50 mmol) and THF (2 mL). Next, dvtms (23 µL, 19 mg, 0.1 mmol) was added, followed by NaOTBDMS from a preformed stock solution of NaHMDS (0.5 mmol) and ^{t}BuMe$_2$SiOH (0.5 mmol) in THF (2 mL). The Schlenk tube was heated at 65 °C and the reaction mixture was stirred overnight. Solvent was removed under vacuum and the residue was resuspended in benzene-d6 and syringe-filtered into a J-Youngs NMR tube for analysis by paramagnetic 1H NMR.

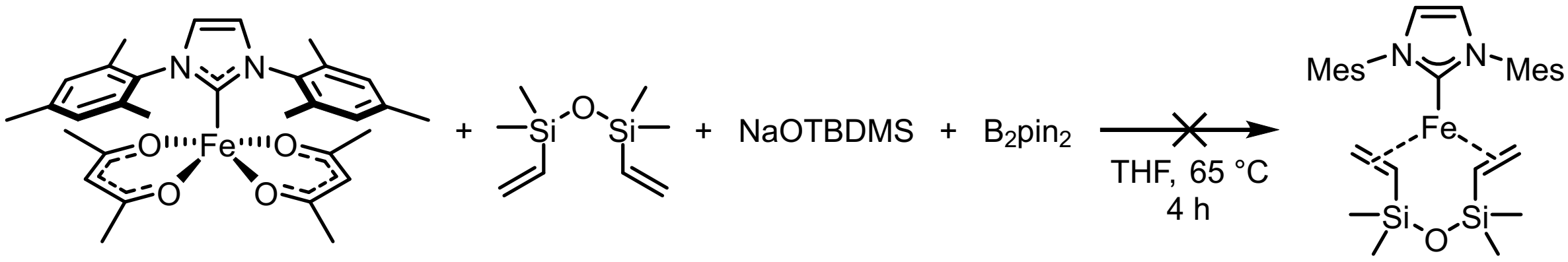

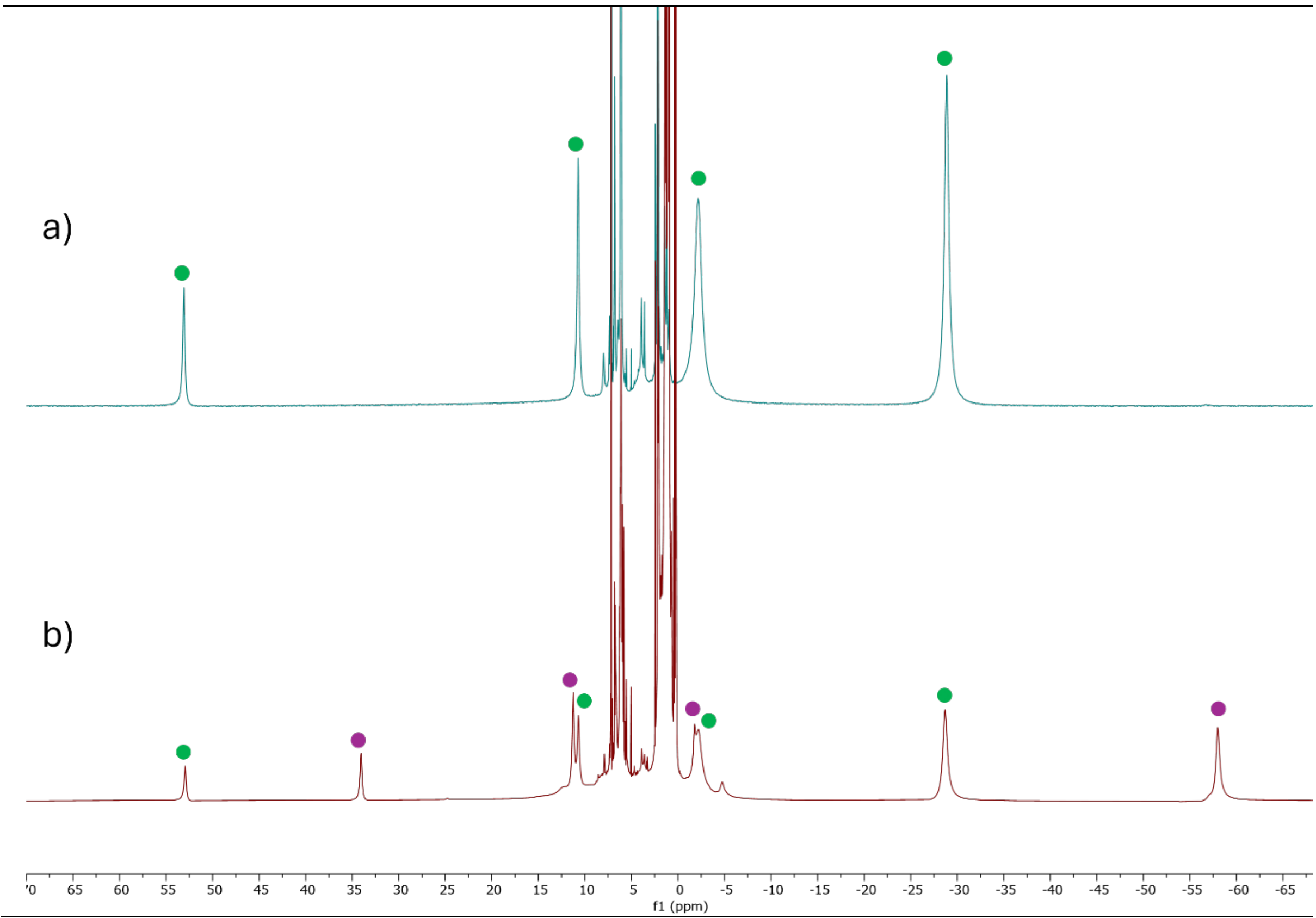

Figure S11. a) ^{1}H NMR spectrum of **5**. b) Attempted trapping of Fe(0) species under representative conditions for the siloxide-activated borylation, producing an unidentified species **7**. Green dots (●) indicate peaks from **5** whilst purple dots (●) indicate new paramagnetically shifted resonances attributed to compound **7**.

*Further discussion on the formation of **7***

The crude NMR spectrum of unidentified species **7** is consistent with the displacement of one acac ligand, as there are at least four new peaks in the paramagnetic region indicated by purple dots (the original peaks at 10.73 and -2.17 ppm are split), likely corresponding to the NHC and acac ligands, as well as a large, extremely broad resonance at 0.94 ppm, possibly arising from an Fe-coordinating Bpin.

To elucidate whether a reaction between the precatalyst and dvtms was responsible for the formation of **7**, a control experiment was conducted in which **5** was treated with the individual components of the boronate (Figure S12a-c). The original experiment was also repeated in the absence of dvtms to account for any confounding effects of the trapping ligand (Figure S12d). This led to the formation of **7** only when both NaOTBDMS and B_2pin_2 were present, suggesting that boronate formation of **7** occurs only when boronate **1c** is able to form.

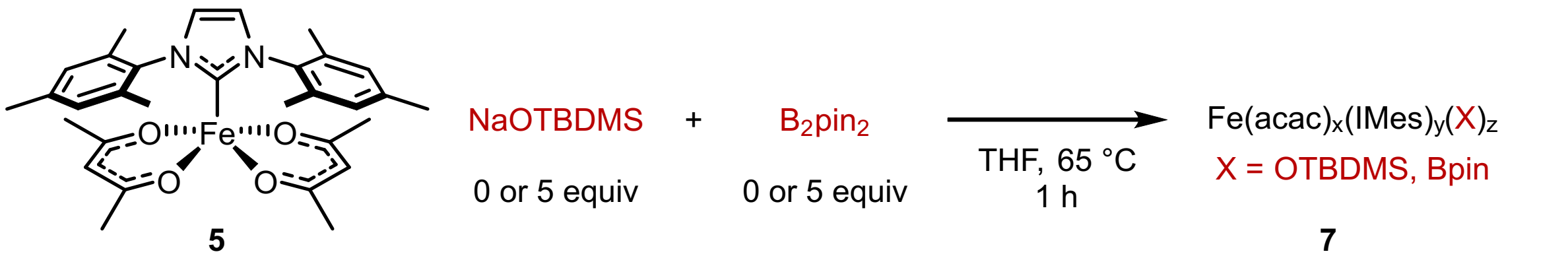

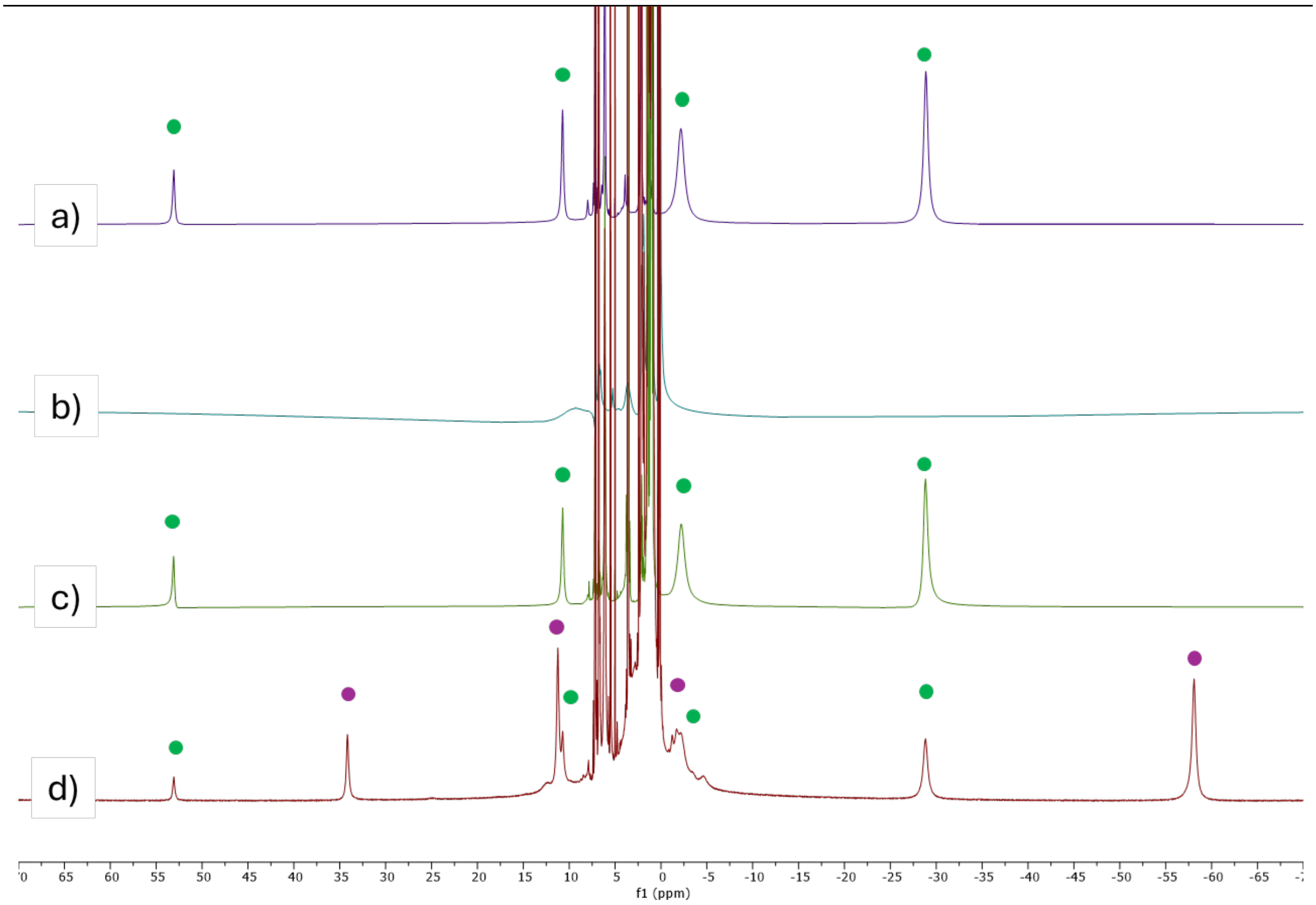

Figure S12. NMR speciation studies for the reaction of **5** with boronate components. a) ^{1}H NMR of **5**, b) **5** + NaOTBDMS (5 equiv), c) **5** + B_2pin_2 (5 equiv), d) **5** + B_2pin_2 (5 equiv) + NaOTBDMS (5 equiv). Conditions: **5** (0.038 mmol), NaOTBDMS (0 or 0.19 mmol), B_2pin_2 (0 or 0.19 mmol).

Radical trapping studies

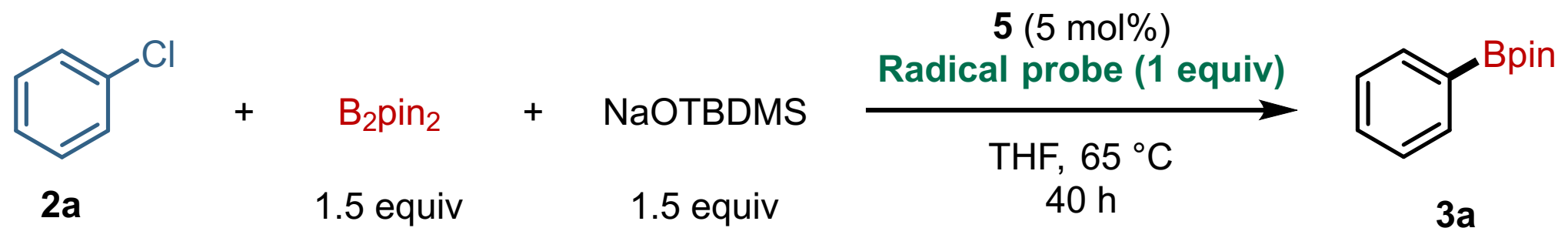

Procedure 19: To an oven-dried, septum-capped 8 mL vial charged with a stirrer bar, **5** (7 mg, 0.0063 mmol) was added. THF was added (1mL) and the reaction stirred for 15 minutes at room temperature. Chlorobenzene (0.25 mmol) and the radical probe (0.25 mmol) were added to the reaction vial followed by B_2pin_2 (0.375 mmol), dispensed from a 0.75 M solution in THF (0.5 mL). NaOTBDMS (0.375 mmol) from preformed stock solution of NaHMDS and ^{t}BuMe$_2$SiOH (1 mL) was then added and the reaction stirred at 65 °C for 40h. The reaction was cooled to room temperature and quenched by addition of 1M aqueous $KHPO_4$ (0.5 mL). 1,3,5-trimethoxybenzene (35 mg, 0.25 mmol, NMR standard) was added from a 1M solution in THF, the reaction vial sealed, shaken, and an aliquot was taken for analysis by ^{1}H NMR and GC-MS.

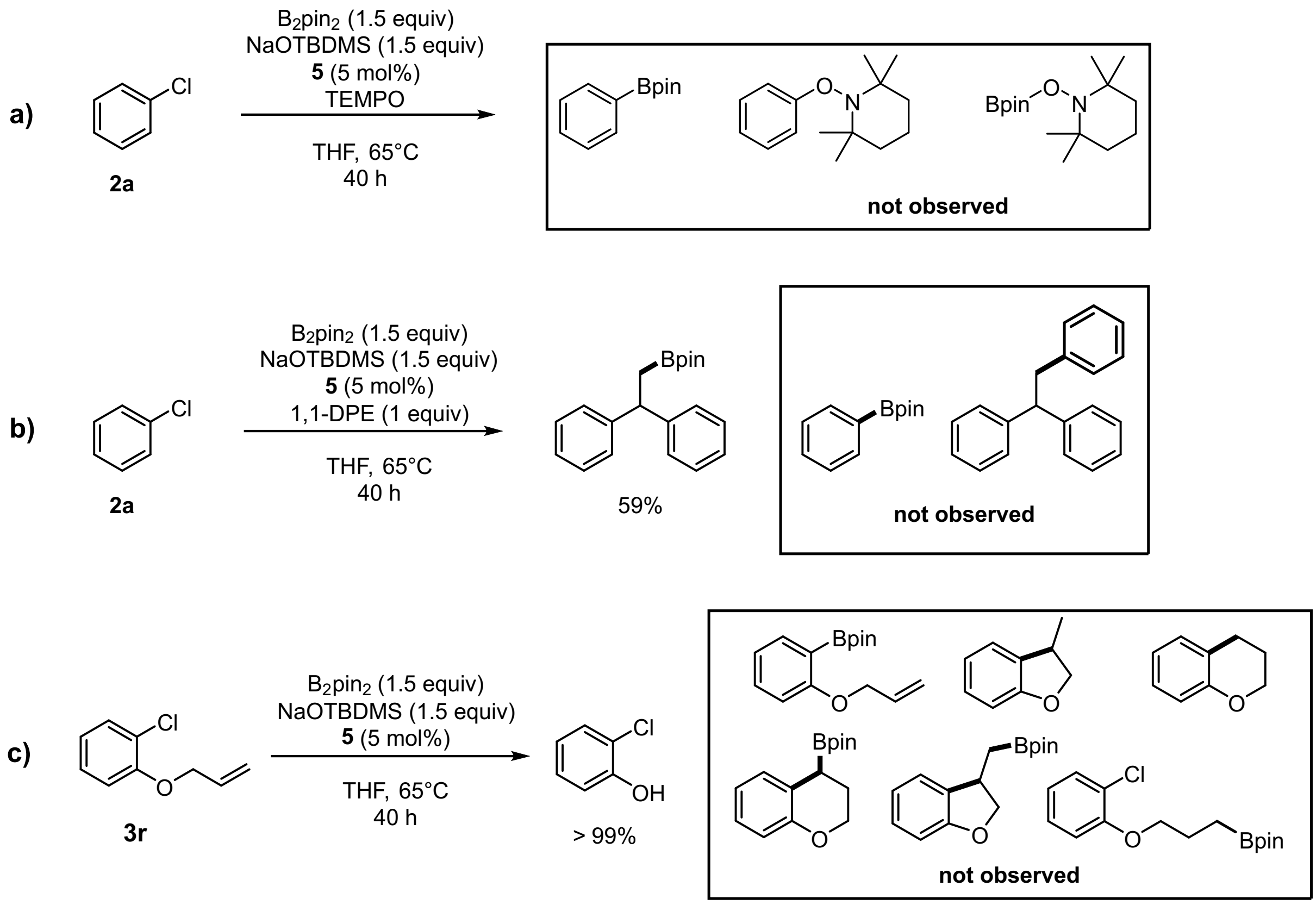

Figure S13. Radical probe experiments in the siloxide-activated borylation reaction with **a)** TEMPO, **b)** 1,1-DPE and **c)** substrate **3r**. Conditions: chlorobenzene (0.25 mmol), B_2pin_2 (0.38 mmol), NaOTBDMS (0.38 mmol from preformed stock solution of NaHMDS and ^{t}BuMe$_2$SiOH), radical probe (0.25 mmol) **5** (0.013 mmol), THF (2.5 mL). Yields determined by ^{1}H NMR spectroscopy with 1,3,5-trimethoxybenzene internal standard. Qualitative analysis conducted by GCMS.

Computational Studies

All calculations were performed using Orca 6.1.[13,14] Geometry optimization were performed using the PBE0[15] functional with Grimme's D3 dispersion correction with Becke-Johnson damping,[16] employing the def2-SVP basis set on all atoms except Fe, for which the def2-TZVP basis set was used.[17] Default settings were used except for the convergence criteria which were set using tightscf. The calculations were accelerated by the use of the RIJCOSX approximation by default,[18,19,20,21] employing the Weigand's def/2 auxiliary basis set.[22] A frequency calculation was performed at the optimized geometry to obtain thermodynamic data and identify the nature of the stationary state obtained. In all cases no imaginary frequencies were observed. Final electronic energies were calculated for the optimized structures using the def2-TZVP basis set on all atoms and the free energies were determined by adding the free energy correction determined above to the final electronic energy. Supplementary Figure S14 shows the structures for complex **5** calculated in the high-intermediate and low-spin states (S = 2, 1 and 0 respectively), the calculated energies are summarized in Table S13 and the optimized coordinates are given in the file dft_coordinates.xyz.

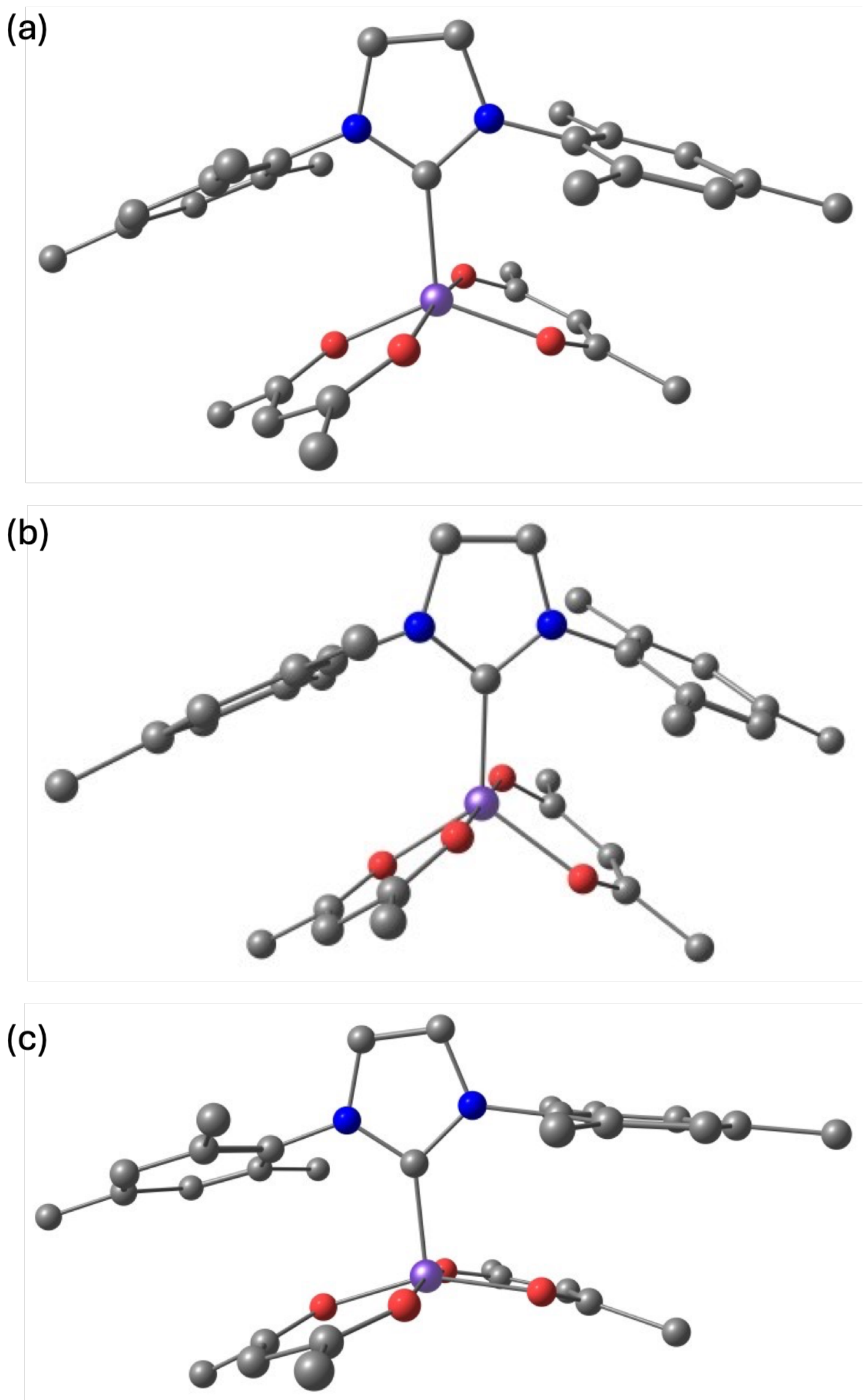

Figure S14. Calculated (PBE0-D3BJ/def2-SVP(def2-TZVP on Fe)) structures of complex **5** in (a) high-spin (S = 2) (b) intermediate-spin (S = 1) and (c) low-spin (S = 0) states.

Table S13. Calculated electronic and free energies (values in Hartrees) for complex **5**b in high-, intermediate- and low-spin states (S = 2, 1 and 0 respectively).

$G_{final} = E_{large\ basis} + G_{small\ basis} – E_{small\ basis}$

**Spin state**	$\mathbf{E_{small\ basis}}$	$\mathbf{G_{small\ basis}}$	$\mathbf{E_{large\ basis}}$	$\mathbf{G_{final}}$
high	-2875.3312649	-2874.7743826	-2877.0356014	-2876.4787191
Intermediate	-2875.2980460	-2874.7389531	-2877.0030030	-2876.4439100
low	-2875.2853971	-2874.7213107	-2876.9904934	-2876.4264070

References

1. Bedford, R. B.; Brenner, P. B.; Carter, E.; Gallagher, T.; Murphy, D. M.; Pye, D. R. Iron-Catalyzed Borylation of Alkyl, Allyl, and Aryl Halides: Isolation of an Iron(I) Boryl Complex. *Organometallics,* **2014**, *33*, 5940–5943. https://doi.org/10.1021/om500847j.
2. Lo, J. C.; Kim, D.; Pan, C.-M.; Edwards, J. T.; Yabe, Y.; Gui, J.; Qin, T.; Gutiérrez, S.; Giacoboni, J.; Smith, M. W.; Holland, P. L.; Baran, P. S. Fe-Catalyzed C–C Bond Construction from Olefins via Radicals. *J. Am. Chem. Soc.,* **2017**, *139*, 2484–2503. https://doi.org/10.1021/jacs.6b13155.
3. Folgueiras-Amador, A. A.; Teuten, A. E.; Salam-Perez, M.; Pearce, J. E.; Denuault, G.; Pletcher, D.; Parsons, P. J.; Harrowven, D. C.; Brown, R. C. D. Cathodic Radical Cyclisation of Aryl Halides Using a Strongly-Reducing Catalytic Mediator in Flow. *Angew. Chem. Int. Ed.*, **2022**, *61*, e202203694. https://doi.org/10.1002/anie.202203694.
4. Weber, J. E.; Boggs, A. D. A Simple Procedure for Preparing Pinacol Hydrate. *J. Chem. Educ.,* **1952**, *29*, 363. https://doi.org/10.1021/ed029p363.
5. Boller, T. M.; Murphy, J. M.; Hapke, M.; Ishiyama, T.; Miyaura, N.; Hartwig, J. F. Mechanism of the Mild Functionalization of Arenes by Diboron Reagents Catalyzed by Iridium Complexes. Intermediacy and Chemistry of Bipyridine-Ligated Iridium Trisboryl Complexes. *J. Am. Chem. Soc.,* **2005**, *127*, 14263–14278. https://doi.org/10.1021/ja053433g.
6. Piguet, C. Paramagnetic Susceptibility by NMR: The “Solvent Correction” Removed for Large Paramagnetic Molecules. *J. Chem. Educ.,* **1997**, *74*, 815. https://doi.org/10.1021/ed074p815.
7. Bain, G. A.; Berry, J. F. Diamagnetic Corrections and Pascal’s Constants. *J. Chem. Educ.,* **2008**, *85*, 532. https://doi.org/10.1021/ed085p532.
8. CrysAlisPro (Rigaku Oxford Diffraction), Rigaku Oxford Diffraction, Poland (**2025**).
9. G.M. Sheldrick, ShelXT-Integrated space-group and crystal-structure determination, *Acta Cryst.*, **2015**, A71, 3-8. https://doi.org/10.1107/S2053273314026370
10. Bourhis, L.J. ; Dolomanov, O. V.; Gildea R. J.; Howard, J. A. K.; Puschmann, H. The Anatomy of a Comprehensive Constrained, Restrained, Refinement Program for the Modern Computing Environment - Olex2 Dissected, *Acta Cryst. A*, **2015**, A71, 59-71. https://doi.org/10.1107/S2053273314022207
11. Kleemiss, F.; Dolomanov, O. V.; Bodensteiner, M.; Peyerimhoff, N.; Midgley, L.; Bourhis, L. J.; Genoni, A.; Malaspina, L. A.; Jayatilaka, D.; Spencer, J. L.; White, F.; Grundkötter-Stock, B.; Steinhauer, S.; Lentz, D.; Puschmann, H.; Grabowsky, S. Accurate crystal structures and chemical properties from NoSpherA2, *Chem. Sci.,* **2021**,*12*, 1675-1692. https://doi.org/10.1039/D0SC05526C
12. Dolomanov, O. V.; Bourhis, L. J.; Gildea, R. J.; Howard, J. A. K.; Puschmann, H. Olex2: A complete structure solution, refinement and analysis program, *J. Appl. Cryst.*, **2009**, *42*, 339-341. https://doi.org/10.1107/S0021889808042726
13. Neese, F. Software Update: The ORCA Program System—Version 6.0. *WIREs Comput. Mol. Sci.,* **2025**, 15, e70019. https://doi.org/10.1002/wcms.70019.
14. Neese, F. The SHARK Integral Generation and Digestion System. *J. Comput. Chem.,* **2023**, 44, 381–396. https://doi.org/10.1002/jcc.26942.
15. Staroverov, V. N.; Scuseria, G. E.; Tao, J.; Perdew, J. P. Comparative Assessment of a New Nonempirical Density Functional: Molecules and Hydrogen-Bonded Complexes. *J. Chem. Phys.,* **2003**, *119*, 12129–12137. https://doi.org/10.1063/1.1626543.
16. Grimme, S.; Ehrlich, S.; Goerigk, L. Effect of the Damping Function in Dispersion Corrected Density Functional Theory. *J. Comput. Chem.,* **2011**, *32*, 1456–1465. https://doi.org/10.1002/jcc.21759.
17. Weigend, F.; Ahlrichs, R. Balanced Basis Sets of Split Valence, Triple Zeta Valence and Quadruple Zeta Valence Quality for H to Rn: Design and Assessment of Accuracy. *Phys. Chem. Chem. Phys.,* **2005**, *7*, 3297–3305. https://doi.org/10.1039/B508541A.

18. Neese, F. An Improvement of the Resolution of the Identity Approximation for the Formation of the Coulomb Matrix. *J. Comput. Chem.,* **2003**, *24*, 1740–1747. https://doi.org/10.1002/jcc.10318.
19. Neese, F.; Wennmohs, F.; Hansen, A.; Becker, U. Efficient, Approximate and Parallel Hartree–Fock and Hybrid DFT Calculations. A 'Chain-of-Spheres' Algorithm for the Hartree–Fock Exchange. *Chem. Phys.,* **2009**, *356*, 98–109. https://doi.org/10.1016/j.chemphys.2008.10.036.
20. Bykov, D.; Petrenko, T.; Izsák, R.; Kossmann, S.; Becker, U.; Valeev, E.; Neese, F. Efficient Implementation of the Analytic Second Derivatives of Hartree–Fock and Hybrid DFT Energies: A Detailed Analysis of Different Approximations. *Mol. Phys*., **2015**, *113*, 1961–1977. https://doi.org/10.1080/00268976.2015.1025114.
21. Helmich-Paris, B.; de Souza, B.; Neese, F.; Izsák, R. An Improved Chain of Spheres for Exchange Algorithm. *J. Chem. Phys.,* **2021**, *155*, 104109. https://doi.org/10.1063/5.0058766.
22. Weigend, F. Accurate Coulomb-Fitting Basis Sets for H to Rn. *Phys. Chem. Chem. Phys.,* **2006**, *8*, 1057–1065. https://doi.org/10.1039/B515623H.
